# Strains, functions and dynamics in the expanded Human Microbiome Project

**DOI:** 10.1038/nature23889

**Published:** 2017-09-20

**Authors:** Jason Lloyd-Price, Anup Mahurkar, Gholamali Rahnavard, Jonathan Crabtree, Joshua Orvis, A. Brantley Hall, Arthur Brady, Heather H. Creasy, Carrie McCracken, Michelle G. Giglio, Daniel McDonald, Eric A. Franzosa, Rob Knight, Owen White, Curtis Huttenhower

**Affiliations:** 1 Biostatistics Department, Harvard T. H. Chan School of Public Health, Boston, 02115 Massachusetts USA; 2 The Broad Institute, Cambridge, 02142 Massachusetts USA; 3 Institute for Genome Sciences, University of Maryland School of Medicine, Baltimore, 21201 Maryland USA; 4 Department of Pediatrics, University of California San Diego, La Jolla, 92093 California USA; 5 Department of Computer Science & Engineering, University of California San Diego, La Jolla, 92093 California USA

**Keywords:** Microbial ecology, Microbial ecology, Metagenomics, Microbiome

## Abstract

The characterization of baseline microbial and functional diversity in the human microbiome has enabled studies of microbiome-related disease, diversity, biogeography, and molecular function. The National Institutes of Health Human Microbiome Project has provided one of the broadest such characterizations so far. Here we introduce a second wave of data from the study, comprising 1,631 new metagenomes (2,355 total) targeting diverse body sites with multiple time points in 265 individuals. We applied updated profiling and assembly methods to provide new characterizations of microbiome personalization. Strain identification revealed subspecies clades specific to body sites; it also quantified species with phylogenetic diversity under-represented in isolate genomes. Body-wide functional profiling classified pathways into universal, human-enriched, and body site-enriched subsets. Finally, temporal analysis decomposed microbial variation into rapidly variable, moderately variable, and stable subsets. This study furthers our knowledge of baseline human microbial diversity and enables an understanding of personalized microbiome function and dynamics.

**Supplementary information:**

The online version of this article (doi:10.1038/nature23889) contains supplementary material, which is available to authorized users.

## Abstract (web summary)

Updates from the Human Microbiome Project analyse the largest known body-wide metagenomic profile of human microbiome personalization.

**Supplementary information:**

The online version of this article (doi:10.1038/nature23889) contains supplementary material, which is available to authorized users.

## Delving deeper into the human microbiome

The National Institutes of Health Human Microbiome Project, published in 2012, provided a broad overview of the baseline microbiome in healthy individuals using samples from 18 different body sites. In this second installment, the authors expand this dataset with new whole-metagenome sequences and additional time points to assess the diversity and spatiotemporal distributions of the microbiota at six of these body sites. Using a combination of strain profiling, species-level metagenomic functional profiling and longitudinal analyses, this study delivers deeper insights into human microbial communities and provides an important resource for understanding what constitutes a 'healthy' microbiota.

**Supplementary information:**

The online version of this article (doi:10.1038/nature23889) contains supplementary material, which is available to authorized users.

## Main

The human microbiome is an integral component in the maintenance of health^1,2^ and of the immune system^3,4^. Population-scale studies have aided in understanding the functional consequences of its remarkable inter-individual diversity, the earliest of which include MetaHIT^5,6^ and the Human Microbiome Project^1^ (referred to here as HMP1). Studies continue to focus on the gut^7,8,9^, with fewer population-scale cohorts investigating vaginal^10^, oral^11^, or skin^12^ microbial communities. HMP1 remains the largest body-wide combined amplicon and metagenome survey of the healthy microbiome to date.

**Extended Data Figure 1:**
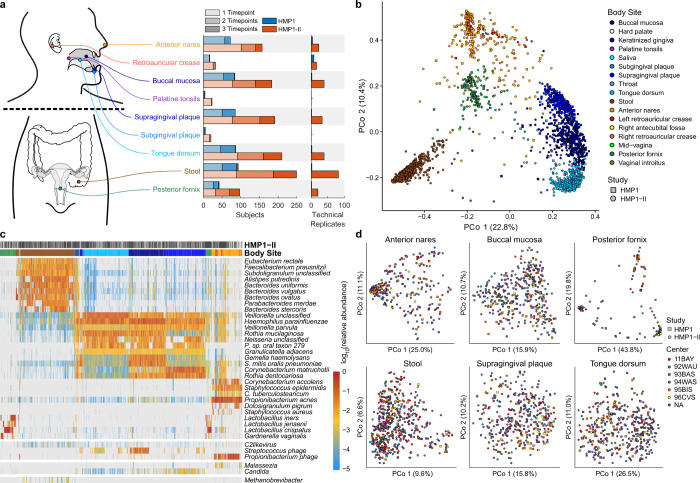
Extended body-wide metagenomic taxonomic profiles in HMP1-II. **a**, The combined HMP1-II datasets include a total of 2,355 metagenomes (724 previously published and 1,631 new, including 252 technical replicates). These span the project’s six targeted body sites (anterior nares, buccal mucosa, supragingival plaque, tongue dorsum, stool, and posterior fornix) in addition to at least 20 samples each from 3 additional sites, of the 18 total sampled sites: retroauricular crease, palatine tonsils, and subgingival plaque. Metagenomes are now available for at least one body site for a total of 265 individuals. **b**, PCoA using Bray–Curtis distances among all microbes at the species level. **c**, Relative abundances of the most prevalent and abundant microbes (bacterial, viral, eukaryotic, and archaeal) among all body sites, as profiled by MetaPhlAn2^20^. Prevalent eukaryotic microbes are shown at the genus level. **d**, Taxonomic profiles do not vary more between sequencing centres, batches, or clinical centres than they do among individuals within body sites. Ordinations show Bray–Curtis principal coordinates of species-level abundances at each body site. Within-site ecological structure is as expected^1^, with no divergence associated with technical variables along the first two ordination axes.

Here we report on an expanded dataset from the HMP (HMP1-II), consisting of whole-metagenome sequencing (WMS) of 1,631 new samples from the HMP cohort^13^ (for a total of 2,355; Extended Data Fig. 1a; Extended Data Table 1a; Supplementary Table 1). New samples greatly expand the number of subjects with sequenced second and third visits, and primarily target 6 body sites (from 18 total sampled): anterior nares, buccal mucosa, supragingival plaque, tongue dorsum, stool, and posterior fornix. After quality control (Methods), the dataset consisted of 2,103 unique metagenomes and 252 technical replicates, which were used in all the following analyses. Profiles, raw data, and assemblies are publicly available at http://hmpdacc.org (Extended Data Table 1b) and https://aws.amazon.com/datasets/human-microbiome-project/.

## Body-wide strain diversity and ecology

**Figure 1:**
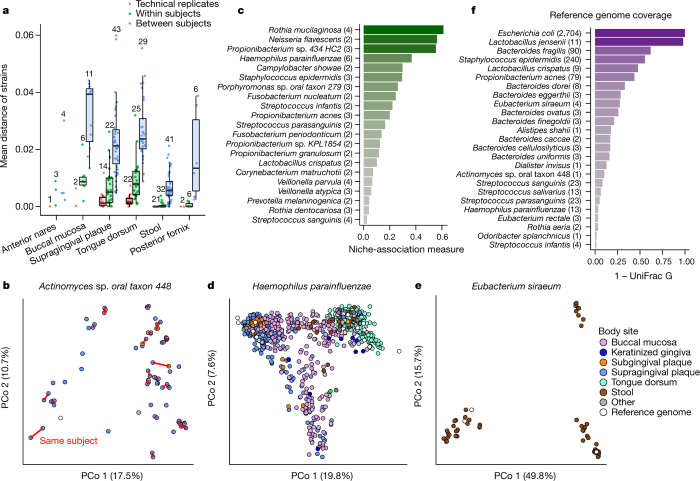
Personalization, niche association, and reference genome coverage in strain-level metagenomic profiles. **a**, Mean phylogenetic divergences^17^ between strains of species with sufficient coverage at each targeted body site (minimum 2 strain pairs). **b**, Individuals tended to retain personalized strains, as visualized by a principal coordinates analysis (PCoA) plot for *Actinomyces* sp. oral taxon 448, in which lines connect samples from the same individual. **c**, Quantification of niche association (Methods; only species with sufficient coverage in at least five samples at two or more body sites). Higher values indicate greater phylogenetic separation between body sites. **d**, PCoA showing niche association of *Haemophilus parainfluenzae*, showing subspecies specialization to three different body sites. **e**, PCoA for *Eubacterium siraeum*. **f**, Coverage of human-associated strains by the current 16,903 reference genome set (Methods). Top 25 species by mean relative abundance when present (>0.1% relative abundance) are shown (minimum prevalence of 50 samples). Sample counts in Supplementary Table 2, and distance matrices are available from Extended Data Table 1b. PowerPoint slide

**Extended Data Figure 2:**
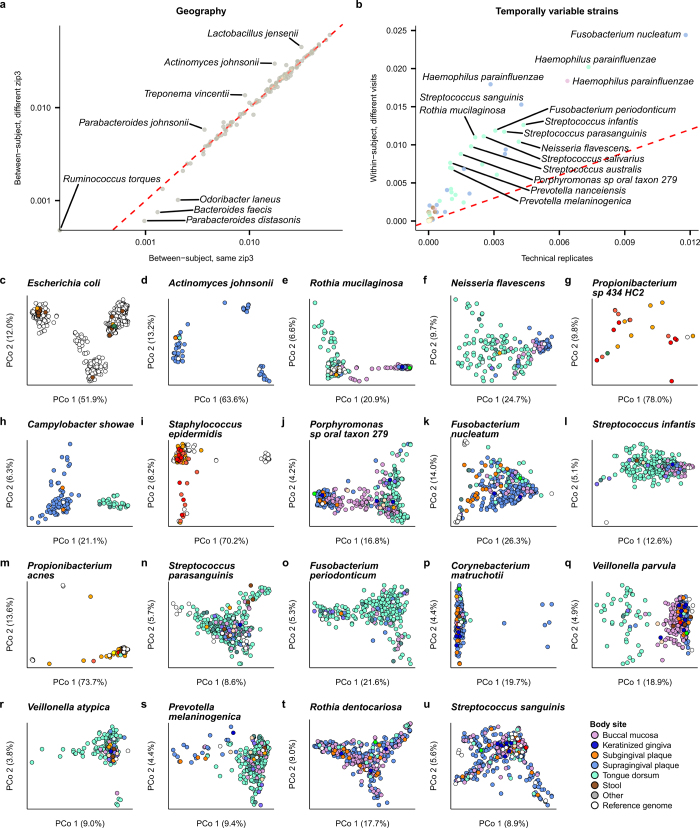
Geographic, temporal, and biogeographic strain variation. **a**, Mean distance (Kimura two-parameter) among strains from subjects either within or between three-digit zip codes (the finest degree of geographic information available). Data and sample sizes are in Supplementary Table 2. **b**, Mean strain divergences between different visits for the same subject and body site compared to the mean distance between the same visits for the same subject and body site (technical replicates) for each species. **c**–**u**, PCoA plots based on the Kimura two-parameter distance^17^ are shown for *Escherichia coli* (**c**), *Actinomyces johnsonii* (**d**), and all species (that is, those shown Fig. 1b ; **e**–**u**), sorted in descending order of their niche-association score (Methods). Distance matrices used to generate these PCoAs are publicly available (Extended Data Table 1b).

The diversity and spatiotemporal distributions of strains were first investigated using StrainPhlAn^14^ (Fig. 1), which identifies the dominant haplotype (‘strain’) of each sufficiently abundant species in a metagenome (Methods, Supplementary Table 2). Most previous culture-independent strain surveys have targeted only the gut^15,16^, and body-wide phylogenetic distances (quantified using the Kimura two-parameter distance^17^) suggest that all other habitats possess greater strain diversity (Fig. 1a). Consistent with previous observations^15,18^, strain profiles were stable over time, with differences over time consistently lower than differences between people (Fig. 1a, b). Nevertheless, technical differences were even lower, indicating a baseline level of intra-individual strain variation over time (Extended Data Fig. 2b).

Several species exhibited differentiation into body site-specific subspecies clades (Fig. 1c; Extended Data Fig. 2e–u), defined here as discrete phylogenetically related clusters of strains, according to a silhouette-based score of niche association (Methods). This is readily visible in extreme cases, such as *Haemophilus parainfluenzae* (Fig. 1d), in which distinct subspecies clades are apparent in the supragingival plaque, buccal mucosa, and tongue dorsum. Other species with notable site-specific subspecies clades included *Rothia mucilaginosa*, *Neisseria flavescens*, and a *Propionibacterium* species. Some species did not sub-speciate within body sites, but instead specialized in clades differing among individuals (for example, *Eubacterium siraeum* (Fig. 1e), or *Actinomyces johnsonii* (Extended Data Fig. 2d)); others showed no discrete subspecies phylogenetic structure at all in this population (for example, *Streptococcus sanguinis*, Extended Data Fig. 2u). Interestingly, no subspecies clades were found to be specific to either of the two cities in the study (Extended Data Fig. 2a), although geographically localized subspecies population structure has been observed in cohorts with greater geographic range^15^.

Culture-independent strain profiling, in combination with the 16,903 NCBI isolate genomes used as references in this analysis^19^, provided a new quantification^20^ of how well covered human microbial diversity is by these references (Fig. 1f). Well-sequenced species such as *Escherichia coli* (Extended Data Fig. 2c) and the lactobacilli showed little divergence from reference isolates. However, many prevalent and abundant species in the body-wide microbiome diverged markedly from the closest available reference genomes. Notable clades lacking isolate genomes representative of those in the microbiome included *Actinomyces* (Fig. 1b), *Haemophilus parainfluenzae* (Fig. 1d), *Eubacterium rectale*, and several *Streptococcus* and *Bacteroides* species, and these represent priority targets for isolation.

Owing to improvements in methodology and reference genomes, new species-level taxonomic profiling included eukaryotes, viruses, archaea, and an additional 54 bacterial species in these metagenomes relative to HMP1 data^1^. The latter contained prevalent bacteria such as *Bacteroides dorei*, *Bacteroides fragilis*, *Alistipes finegoldii*, *Alistipes onderdonkii*, and unclassified species of *Subdoligranulum* and *Oscillibacter*. The former included *Methanobrevibacter*, *Malassezia*, and *Candida* (Extended Data Fig. 1c), as well as several viruses: *Propionibacterium* phage in the anterior nares, *Streptococcus* phages in oral sites, and a *Lactococcus*-targeting C2-like virus in stool. Searching for co-occurrence patterns with non-bacterial species (Fisher’s exact test, presence/absence threshold of 0.1% relative abundance; Supplementary Table 3), we found that *Methanobrevibacter smithii* tended to co-occur with several Clostridiales species in the gut, including members of *Ruminococcus*, *Coprococcus*, *Eubacterium*, and *Dorea* (false discovery rate (FDR) less than 0.1), reinforcing previous observations^21^ and consistent with co-occurrence patterns of methanogens and clostridia in lean versus obese individuals^22^. Prominent *Streptococcus* phages, which were the most abundant species in the oral cavity, also co-occur with numerous *Streptococcus* species in oral sites, suggesting that the virus predominantly exists as a prophage, as observed previously^23^.

## Core pathways of the human microbiome

**Figure 2:**
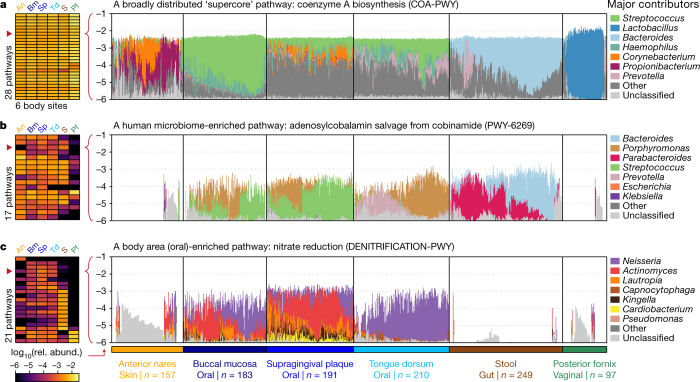
Core and distinguishing functions of human body site microbiomes. **a**, In total, 28 metabolic pathways were core at all 6 major body sites (‘supercore’ pathways). An, anterior nares; Bm, buccal mucosa; Pf, posterior fornix; S, stool; Sp, supragingival plaque; Td, tongue dorsum. Two supercore pathways and **b**, 17 additional pathways were core in multiple body areas and enriched among human-associated taxa (‘human microbiome-enriched’ pathways). **c**, 21 pathways were considerably more abundant at 1 body site than at sites from all other body areas (‘body site-enriched’ pathways). Heat map values reflect the first quartile of relative abundance (heat maps are expanded in Extended Data Fig. 3). In pathway bar plots, total (community) abundance is log-scaled, and the contributions of the top seven genera are proportionally scaled within the total. ‘Other’ encompasses contributions from additional, known genera; ‘unclassified’ encompasses contributions of unknown taxonomy. PowerPoint slide

**Extended Data Figure 3:**
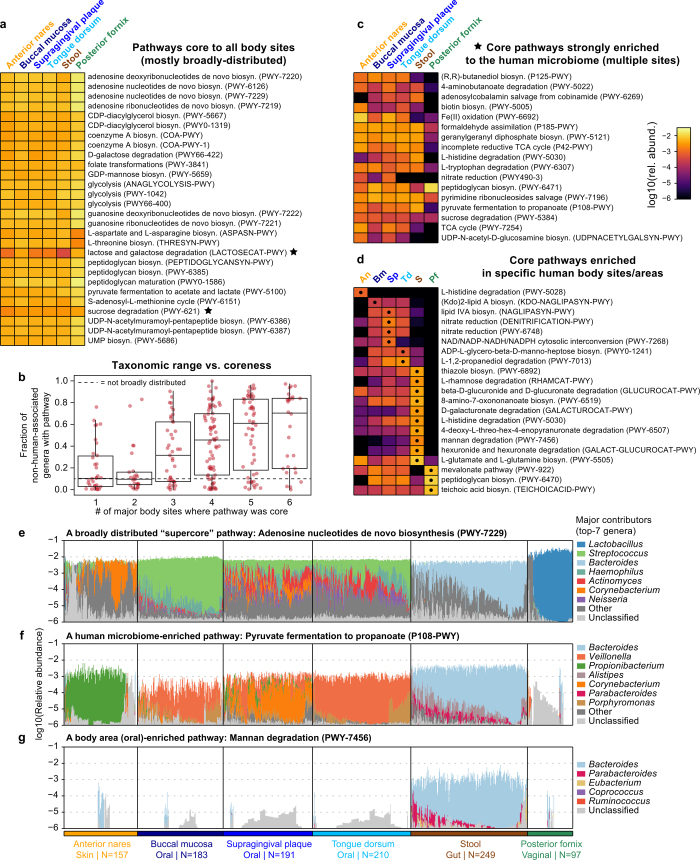
Core and distinguishing functions of human body site microbiomes. This figure extends Fig. 3, with additional details and examples. **a**, 28 metabolic pathways were core (>75% prevalent) at all major body sites. We refer to these as ‘supercore’ pathways. **b**, Pathways core to greater numbers of body sites tended to have broader taxonomic ranges, with supercore pathways among the most broadly distributed (Tukey boxplots). **c**, 19 pathways (including two supercore pathways, starred in **a**) were core in multiple body areas and specifically enriched among taxa inhabiting the human microbiome (annotated to <10% of non-human-associated genera). Human-microbiome-enriched pathways include specific MetaCyc-defined variants of more broadly defined or distributed processes, for example, peptidoglycan biosynthesis (PWY-6471). **d**, ‘Site-enriched’ pathways are considerably more abundant at one body site than at sites from all other body areas. Black dots indicate the site where each site-enriched pathway achieved its peak abundance. Heat map values reflect the first quartile of relative abundance in a particular body site (coordinated with the percentile cutoff for a core pathway). **e**–**g**, Additional examples of the three pathway classes enumerated in **a**, **c**, and **d**, respectively. In each example, total (community) abundance is log-scaled, and the contributions of the top seven genera are proportionally scaled within the community total. ‘Other’ encompasses pathway contributions from genera outside of the top seven, and ‘unclassified’ encompasses pathway contributions from unidentified members of the community.

Strong prevalence (‘coreness’) of a molecular function across niche-related microbial communities can be explained by either broad taxonomic distribution of the function (as in the case of essential housekeeping functions), or specific enrichment of the function among taxa inhabiting that niche (possibly because the function is selectively advantageous there). We investigated these mechanisms among core metabolic pathways of the human microbiome by functionally profiling all HMP1-II samples using the program HUMAnN2^24^ (Fig. 2; Extended Data Fig. 3, Supplementary Table 4, Methods). We focused on 1,087 metagenomes representing the first sequenced visit from each subject at the 6 targeted body sites. We considered a pathway to be ‘core’ to a specific body site (niche) if it was confidently detected in more than 75% of individuals with strong taxonomic attribution and a taxonomic range consistent with the human microbiome. From a starting set of 857 quantifiable pathways from the MetaCyc^25^ database, we detected 950 instances of a pathway being core to a body site: 258 pathways were core to at least 1 body site, 176 were core to body sites from multiple body areas, and 28 were core to all 6 targeted body sites (Fig. 2a; Extended Data Fig. 3a). For convenience, we refer to these classes as core pathways, multicore pathways, and supercore pathways, respectively.

To distinguish between coreness resulting from broad taxonomic distribution versus niche-specific enrichment, we classified pathways according to their taxonomic range (quantified as the fraction of non-human-associated genera to which they were annotated in the BioCyc database collection). While the majority of pathways were annotated to fewer than 10% of genera, core pathways were annotated to 34% of genera, multicore pathways to 48%, and supercore pathways to 70% (median values; all enrichments over background had *P* < 0.001 by Wilcoxon rank-sum tests). Thus, coreness to a human body site is often associated with broad taxonomic distribution, and pathways that are core to more body sites tend to be more broadly distributed (Spearman’s *r* = 0.40; *P* < 0.001; Extended Data Fig. 3b). Extreme examples included biosynthesis of coenzyme A biosynthesis (see Fig. 2a) and of adenosine nucleotides (Extended Data Fig. 3e)—two ‘housekeeping’ functions that are broadly distributed across not only the human microbiome, but also across all microbial life^26,27^. While we lack dispensability information for entire MetaCyc pathways, we found that individually essential gene families were considerably more prevalent than non-essential families across these samples (median 0.94 versus 0.24; Wilcoxon rank-sum test, *P* < 0.001; Methods), consistent with essential functions being core to many body sites.

Conversely, 19 out of the 176 multicore pathways (including 2 supercore pathways) were confidently not broadly distributed, defined conservatively as being annotated to fewer than 10% of non-human-associated genera in BioCyc, and reconstructed from fewer than 10% of pangenomes in the HUMAnN2 database (Extended Data Fig. 3a, 4c). In these cases, coreness to multiple human body areas is better explained by enrichment among human-associated taxa, and may be indicative of functional adaptation to the human host as a broader niche. Notably, of these 19 pathways, 13 (68%) were more than twofold enriched in human-associated genera than in non-human-associated genera in BioCyc, although this was not required by their definition. Human microbiome-enriched pathways included vitamin B_12_ biosynthesis (adenosylcobalamin salvage from cobinamide), a process commonly performed by the microbiota that must be supplemented in germ-free mice (Fig. 2b). Vitamin B_12_ biosynthesis was also core in the oral cavity, where salivary haptocorrin may protect it for later absorption in the small intestine^28^. Fermentation to propionate (a short-chain fatty acid) was also specifically enriched in the oral and gut environments (Extended Data Fig. 3f). Short-chain fatty acids are noteworthy for their proposed role in the maintenance of gut health^29^, whereas their role in the oral cavity is less well studied.

Finally, a number of core pathways were specifically enriched in individual body sites. We identified a single site-enriched core pathway from the anterior nares, seven from the oral body area (notably, there were few that were enriched for a single oral site), ten from stool, and three from the posterior fornix (Extended Data Fig. 3d). Examples of site-enriched pathways included nitrate reduction in the oral cavity (a known oral microbiome process related to nitrate accumulation in saliva^30^; Fig. 2c) and mannan degradation in the gut (mannan is a plant polysaccharide found in human diet^31^; Extended Data Fig. 3g). Such site-enriched pathways are suggestive of functional adaptation by the microbiota to a particular niche within the human body. Hence, whereas many core functions of the human microbiome reflect broadly distributed, globally essential metabolic processes, others are potentially indicative of microbial community adaptation to specific body sites or to the human host in general.

## Characterization of temporal variability

**Figure 3:**
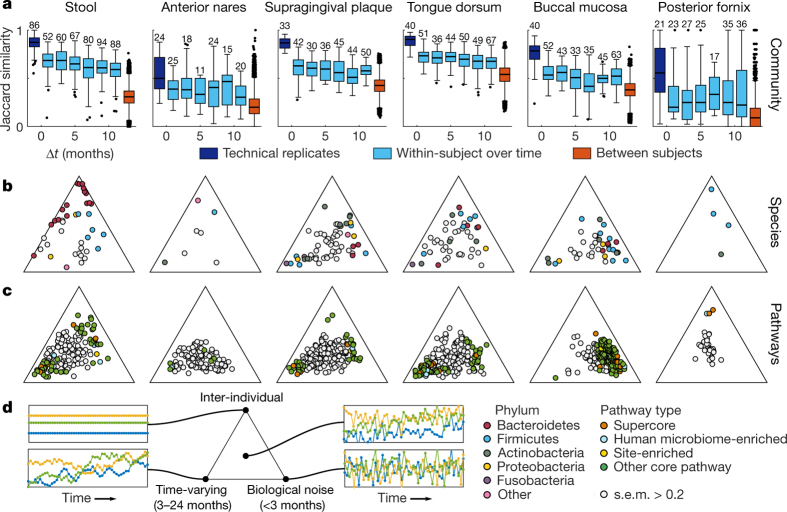
Temporal dynamics of individual species and microbial pathways at each targeted body site. **a**, Jaccard similarity is maximal between technical replicates and decreases with time, although within-subject similarity always exceeds between-subject similarity. **b**, Gaussian process decomposition of the variance in species abundances (each point is one species; filtering criteria in Methods) into three biologically relevant components based on their characteristic timescales (Methods). Technical noise was estimated (Supplementary Table 5) but not visualized. Species with high inference uncertainty (s.e.m. on the ternary diagram > 0.2) are grey and the inference is biased towards the centre of the diagrams (Methods). Labelled version in Extended Data Fig. 5. **c**, Same as **b**, but for abundances of all core pathways. **d**, Illustrative time series showing dynamics at different locations within the ternary plots (Extended Data Fig. 4d–f for real examples). Sample counts in Supplementary Table 1. PowerPoint slide

The new availability of body-wide WMS samples at multiple time points per individual allowed us to characterize further the dynamics of microbial community composition at the species level (Fig. 3). Community-wide species retention rates were comparable to previous observations at all body sites except the posterior fornix^32,33^ (Fig. 3a). To characterize the dynamics of individual species, we developed a Gaussian process model (Methods) that decomposed variability in abundance into four components: constitutive differences between subjects, time-varying dynamics (change measurable at a scale of several months), biological noise (true variation that appears instantaneous relative to our sampling), and technical noise (between technical replicates).

**Extended Data Figure 4:**
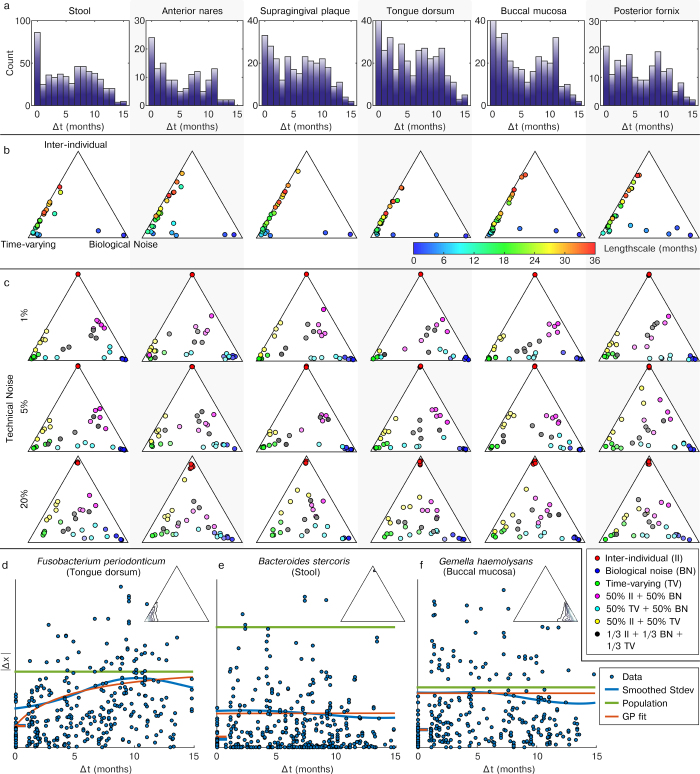
Sampling interval distribution, parameter fits for simulated samples, and examples of microbial species abundance dynamics and corresponding Gaussian process fits. **a**, Distributions of differences in time between samples at each targeted body site. Technical replicates are shown as Δ*t* = 0. **b**, Parameter fits for simulated samples with *U* = 0, *B* = 0, *T* = 0.95, *N* = 0.05, and varying *l* (see Methods). Simulated samples were drawn with the real sample distribution and count from each site, to show how limitations in sampling at certain sites alter the fidelity of the fits. **c**, Parameter fits for five simulated samples with each of the three pure components (coloured red, green and blue), as well as all even mixtures of pairs of them (for example, yellow points are even mixtures of *U* and *T*), and even mixtures of all three (black), for differing levels of technical noise (*N*) and fixed *l* = 0.5. Uncertain inferences are more desaturated. **d**–**f**, Three examples of taxonomic profiles fit with the Gaussian process model are shown on plots designed to allow a direct comparison between the data and the fit Gaussian process, and allow the different dynamics to be visualized despite the limit of only up to three time points per person. Each example was chosen as an exemplar of one of the three non-technical components in the model. Insets denote confidence deciles of the MCMC samples. The abundance of *Fusobacterium periodonticum* in the tongue dorsum shows strong time-varying behaviour (**d**), *Bacteroides stercoris* in stool shows mostly inter-individual differences (**e**), and *Gemella haemolysans* in the buccal mucosa is dominated by biological noise (**f**). The plots show the absolute difference in arcsine square-root transformed microbial abundance (|Δ*x*|) between pairs of samples from the same person against the difference in time between samples (points). A Gaussian-smoothed estimate of the standard deviation of the points is also shown (blue line, bandwidth three months), along with the expected difference from the fit Gaussian process (red line). The standard deviation of differences between technical replicates (points with Δ*t* = 0 months) is also shown as the line stub at the origin, directly visualizing the level of technical noise. Biological noise is visible here as the difference between technical noise and the variance of the remaining points extrapolated to the origin. The time-varying component is visible as a gradual increase in the variance of the differences over time (that is, gradually increasing red and blue lines). Finally, inter-individual differences are visible by comparing the limit of the variance of the data with the variance of differences between subjects (green line).

**Extended Data Figure 5:**
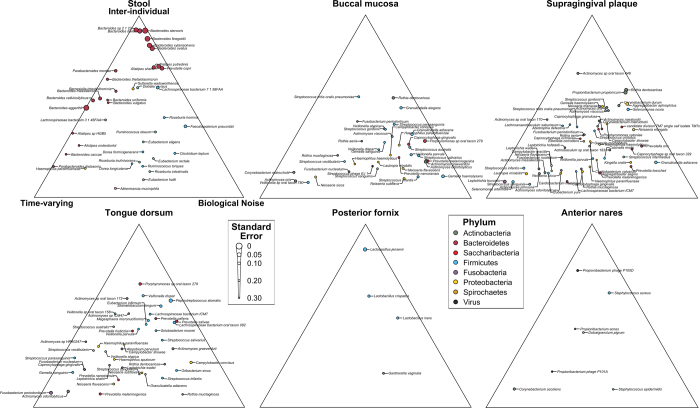
Gaussian process decomposition of temporal variance for metagenomic species abundances. The posterior mean of the decomposition of variance (Methods) is shown for each species (Supplementary Table 5), coloured by phylum. Uncertainty in the estimate was assessed by the square root of the mean squared distance on the ternary plot of MCMC samples from the posterior mean, and is codified with larger points indicating more certain estimates.

This analysis indicated which species at which body sites varied most between individuals, temporally, or rapidly (Fig. 3b, Supplementary Table 5, Extended Data Fig. 4d–f). In the gut, Bacteroidetes species, and in particular the *Bacteroides* genus (Extended Data Fig. 5), exhibited primarily inter-individual variation, whereas Firmicutes were more temporally dynamic within individuals. Species abundances in the oral and skin microbiomes, meanwhile, exhibited greater time-varying dynamics and biological noise overall, and were less personalized, consistent with previous stability assessments^18^. A more detailed look (Extended Data Fig. 5) showed that some species possessed very similar dynamics when detected in multiple body sites (for example, *Rothia dentocariosa*). Others, often those with site-specific subspecies clades analysed above, possessed different dynamics between body sites (for example, *Haemophilus parainfluenzae*). On a broad scale, these species dynamics are in agreement with a previous analysis of whole-community dynamics in the same cohort^34^.

We repeated this Gaussian process analysis to characterize the dynamics of pathway abundances for all core pathways identified above (Fig. 3c, Supplementary Table 5). Pathway abundances at all body sites except the posterior fornix were less personalized than the taxa that encoded them (farther from the inter-individual vertex), consistent with the hypothesis that community assembly is primarily mediated by functional niches rather than a requirement for specific organisms^35,36^. Time-varying pathways were enriched for amino acid biosynthesis (*P* = 0.00025; Wilcoxon rank-sum test), whereas inter-individual pathways were enriched for vitamin B biosynthesis (*P* = 0.00062). By contrast, the vaginal microbiome showed a large personal component, at both the species and pathway levels (all well-fit pathways near the inter-individual vertex), consistent with variation among stable community state types in the vaginal microbiome^37^. Functional dynamics in the gut were relatively slow, possibly reflecting trends in response to long-term factors such as dietary patterns. Conversely, dynamics in oral cavity sites were rapid, in particular in the buccal mucosa, in accordance with the enrichment of the habitat for fast energy harvest and much greater environmental exposure.

## Gene family discovery by assembly

**Figure 4:**
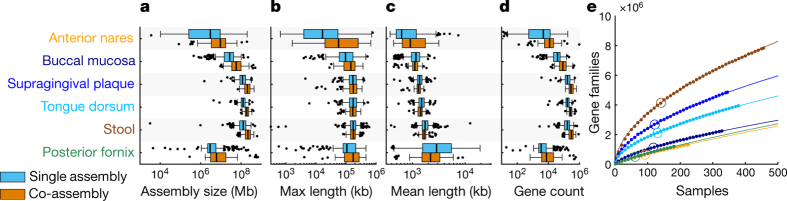
Assembly and annotation of body-wide human microbiomes. **a**–**d**, Tukey boxplots of total assembly size, maximum and average contig lengths, and gene counts for single and co-assemblies (sample sizes in Supplementary Table 1). **e**, Rarefaction curves of gene families (open reading frame (ORF) clusters at 90% sequence similarity) from predicted genes generated from single assemblies for the targeted body sites (points), with a power-law fit (lines). The size of the HMP1 WMS dataset at each body site is also shown (open circles). The rarefaction trajectory was robust to changes in the sequence similarity threshold (for the 188 posterior fornix samples, the number of gene families ranged between only 1,131,796 to 1,271,891 for similarities 70–95%). Colouring is as in axis labels of **a**. Sample counts in Supplementary Table 1. PowerPoint slide

**Extended Data Figure 6:**
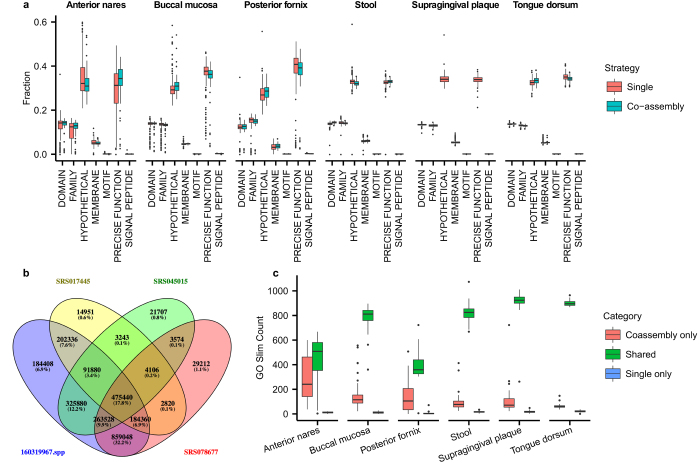
Assembly annotation specificity for single and co-assemblies. **a**, Tukey boxplot of the percentage of proteins in each functional specificity category. **b**, An example Venn diagram for one set of single-sample supragingival plaque assemblies and their combined co-assembly (co-assembly on bottom left), showing counts of shared genes (computed via strict alignment) between all combinations of assemblies; the co-assembly by itself contains 96.9% of all detected genes. **c**, Tukey boxplot of the number of Gene Ontology (GO) terms (generated using a GO Slim with around 1,700 terms) shared between single and co-assemblies, unique to the co-assembly, or unique to one of the single assemblies, generated from a random selection of 250 assemblies across 6 body sites. Co-assemblies capture GO terms that are not in individual assemblies.

We next sought to establish an expanded gene catalogue based on assembly of the expanded set of metagenomes. On the basis of extensive benchmarking, we chose a custom assembly protocol using the IDBA-UD^38^ algorithm (Methods). Compared to the 725 assemblies generated in HMP1^1,13^, this protocol led to improvements in average assembly size, median contig length, and N50 length (Supplementary Table 6). Median metagenome assembly sizes ranged from 2.9 megabases (Mb) for the posterior fornix to 127.6 Mb for stool. To help detect new genes and improve overall assembly quality, we created additional co-assemblies from the combined set of reads from the same individual sampled at the same body site across multiple visits. In total, 406 and 240 co-assemblies were created by combining 2 and 3 visits, respectively (Supplementary Table 6), and the assembly sizes were on average 86% larger than single assemblies: the median assembly size increased from 84.8 Mb to 158.4 Mb, and the median of the maximum contig size in each assembly increased from 152 kilobases (kb) to 167 kb (Fig. 4a–c). Gene finding was performed on contigs using the MetaGeneMark^24^ sequence analysis tool (Fig. 4d; Supplementary Table 7). In co-assemblies, the average number of genes detected increased from 118,177 to 213,741, whereas the mean gene length remained similar (614 compared to 610 nucleotides). Functional assignments were made using Attributor (Methods) based on several sequence-based searches, and classified according to specificity. Approximately 35–45% of genes received specific functional annotations, and around another 30% received annotations at the domain, family, or motif level (Extended Data Fig. 6). In all cases, the number of genes in each specificity category increased in the co-assemblies, although the percentages remained similar. Therefore, although more genes were predicted from the co-assemblies, their annotations are as specific as in single assemblies.

**Extended Data Figure 7:**
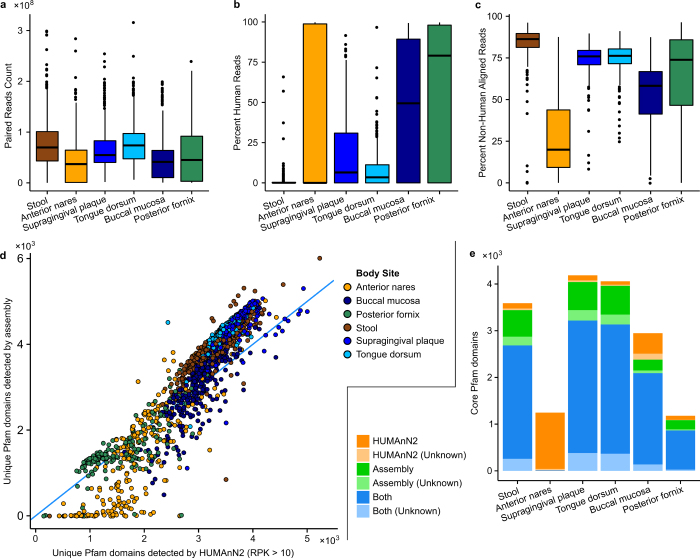
Sequencing statistics and assembly quality assessments. **a**, Tukey boxplots of total raw reads per sample among body sites upon retrieval from the SRA. **b**, Percentages of human reads marked by BMTagger per body site. **c**, Percentages of non-human (bacterial) reads aligned to assemblies showing assembly effectiveness (Methods; read and contig mapping to assemblies and reference genomes). **d**, Comparison of the number of unique Pfam domains detected in each sample by HUMAnN2 and in the assemblies, coloured by body site. Pfam domains in HUMAnN2 were considered ‘detected’ if UniRef50 sequences annotated with the domain were present in a sample at >10 reads per kilobase (around 1× coverage). Pfam domains were detected in the assemblies if they were found on a single contig by Attributor (Methods). **e**, Number of Pfam domains that were detected in at least 75% of samples (core’ domains) by each method, for each targeted body site. Pfams domains are stratified by unknown function.

The number of distinct, well-covered Pfam^39^ domains detected by reference-based versus assembly-based profiling tended to correlate strongly within the same sample (Spearman’s *r* = 0.92; Extended Data Fig. 7d), suggesting that the two methods provide similar relative rankings of community functional diversity. In addition, the two methods tended to co-detect most Pfam domains that were core to a body site (greater than 75% prevalent; Extended Data Fig. 7e). While reference-based profiles called the presence of Pfam domains based on the annotations of characterized proteins, they could be directly detected in assemblies through profile alignment, thus capturing novel sequence diversity. Indeed, assembly tended to detect (median) 19% more Pfam domains per sample than the reference-based approach, which conversely tended to detect established Pfam domains with greater sensitivity. This effect was particularly notable in the anterior nares site, where reduced microbial sequencing depth limited the sensitivity of assembly relative to reference-based profiling.

Compared to external datasets, total non-redundant gene clusters were similar to MetaHIT in the stool^6^ (HMP1-II contained 7,780,363 gene clusters, MetaHIT had 9,879,896); relative to existing moist skin site metagenomes^12^, HMP1-II represented a 780% increase (170,206 gene clusters to 1,326,693). However, even with thousands of deeply sequenced human microbiomes in this study, microbial gene family space is not yet saturated for any of the six examined body sites (Fig. 4e).

## Conclusions

Here we provide and analyse the largest known body-wide metagenomic profile of the human microbiome to date. The associated deep, longitudinal shotgun sequencing has enabled a broad-scale characterization of new aspects of the personalized microbiome. New strain profiling techniques^14^ distinguished temporally stable subspecies population structures for several species, some unique to individuals and others associated with particular body sites. Species with human microbiome strain diversity under-represented in isolate genomes were identified, to be prioritized for isolation and sequencing. New taxonomic profiling resolved co-occurrence patterns between bacterial abundances and several archaea, eukaryotes, and viruses. New functional profiling methods^24^ identified pathways required for microbial colonization of the human body, differentiating those enriched for the human habitat from those universal to microbial life. Gaussian process models characterized microbial and functional variation over time, and identified the composition of the gut community (Bacteroidetes species in particular) as highly personalized compared to other sites. This example implies that the gut Bacteroidetes/Firmicutes balance may not be a defining attribute of an individual’s gut microbiome; instead, individuals carry a ‘personal equilibrium’ among Bacteroidetes, with a group of phylogenetically diverse, temporally variable Firmicutes fluctuating atop this core.

**Extended Data Figure 8:**
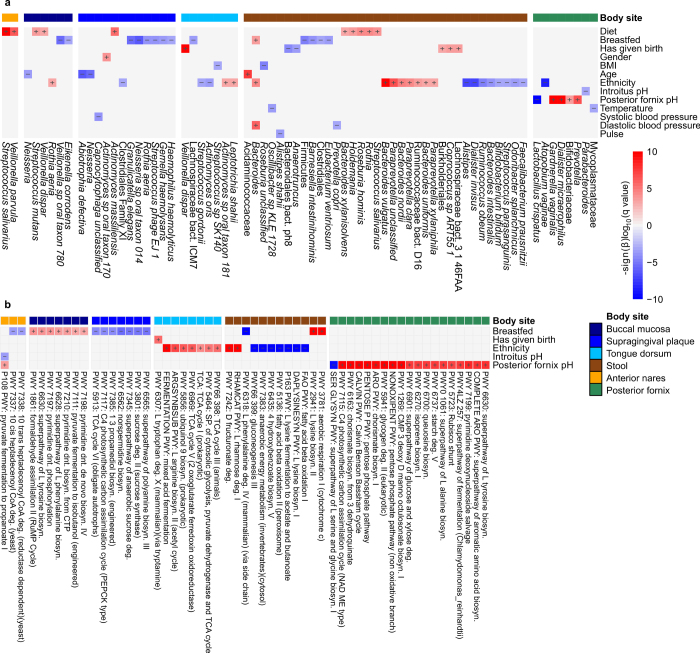
Metagenomic features abundances significantly associated with host phenotype. **a**, **b**, Significant associations of nontrivial effect size (FDR < 0.1 and |*β*| > 0.01) in a multivariate linear model (significance and coefficients in Supplementary Table 5) between taxon abundances (**a**) and pathway abundances (**b**). All detected associations are independent of all other metadata, including whether the subject was breastfed, the subject’s broad dietary characterization, temperature, introitus pH, posterior fornix pH, gender, age, ethnicity, study day processed, sequencing centre, clinical centre, number of quality bases, percentage of human reads, systolic blood pressure, diastolic blood pressure, pulse, whether the subject had given birth, HMP1/HMP1-II, and BMI (group sizes in Extended Data Table 1; see Methods). Non-significant associations here should not be considered evidence of no association.

**Extended Data Figure 9:**
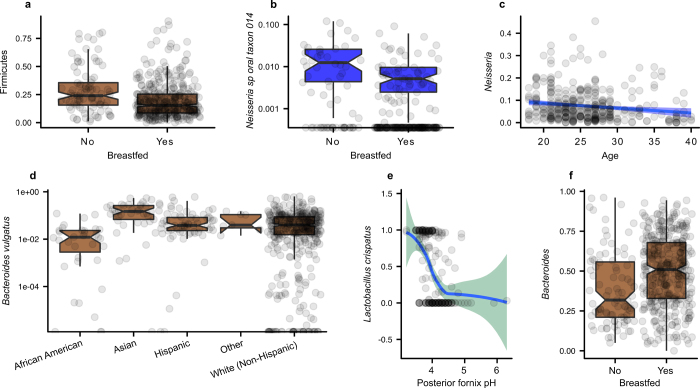
Updated associations in HMP1-II. **a**, HMP cohort subjects reported whether they were breastfed as infants. Remarkably, overall phylum Firmicutes abundances were lower even during adulthood (subjects’ current ages were 18–40) in individuals who had been historically breastfed. **b**, **c**, Differences associated with infant breastfeeding persisted in other clades and body sites, for example, oral *Neisseria* (**b**), although age-linked associations differed among taxa (for example, overall oral *Neisseria* decrease with age) (**c**). **d**–**f**, Examples of associations significant in the original HMP1 metagenome set^1^ that were retained in the larger HMP1-II dataset include: **d**, *Bacteroides vulgatus* in stool is significantly more abundant in Asian people compared to those of other ethnicities. **e**, *Lactobacillus crispatus* in the posterior fornix is negatively associated with vaginal pH. **f**, *Bacteroides* are significantly more abundant in individuals who have been breastfed as infants. Boxplot whiskers are defined by Tukey’s method.

Many key properties of the human microbiome remain to be characterized even in healthy cohorts, in addition to microbiome contributions to disease. Further investigation will be required to determine the functional origins and consequences of subspecies structures identified here. Such structures must also be investigated comprehensively across populations, including variations in geography, genetic background, ethnicity, and environment (for example, outside of the HMP1-II’s North American focus). Notably, the evidence in this study suggests that, even in this relatively homogeneous population with extensive metagenomic sampling, the full complement of extant microbial genes has not yet been sequenced. Similarly, although an updated covariation analysis between metadata and microbial features (Supplementary Note; Extended Data Figs 8 and 9) revealed several novel associations, most variance in the microbiome is not explained by measured covariates. The HMP1-II, for example, did not measure transit time^8^, immune status, or the participants’ detailed diet and pharmaceutical history, limiting our ability to assess these important factors. Finally, our understanding of the dynamics and responses of microbial communities must be expanded from the descriptive models here to include the rapid effects of acute perturbations. For this, studies with longer, more densely sampled time courses in the presence of controlled perturbations will be required, beyond the three time points used here. To rationally repair a dysbiotic microbiome, it is thus necessary to deepen our understanding of the personalized microbiome in human health.

## Methods

### Data reporting

No statistical methods were used to predetermine sample size, as the data included here were derived from biospecimens previously collected during the first wave of Human Microbiome Project studies. As no treatment or phenotype groups were included, no randomization of experiments or blinding were performed.

### HMP1-II samples and metagenomic sequencing

Sample collection, storage, handling, and WMS sequencing were performed as in the HMP1^1^. Details on IRB review, informed consent, subject exclusion criteria, the sampling protocols, and timeline can be found in previous publications^1,13,40^. All metagenomes analysed here were obtained from the SRA after human DNA removal by the SRA using BMTagger (Extended Data Fig. 7a). All SRA native format read files were converted to FASTQ for further analysis using the fastq-dump utility from the SRA SDK toolkit^19^.

### Quality control of nucleotides, reads, and samples

One or more SRA read files from each sample were concatenated per read direction to create a single pair of FASTQ files for each sample. These FASTQs were converted to unaligned BAM using Picard (http://broadinstitute.github.io/picard/) and exact duplicates were removed with a modified version of the Picard EstimateLibraryComplexity module. Finally, all reads were trimmed and length filtered (-q2 -l60) using the trimBWAstyle.usingBam.pl script from the Bioinformatics Core at UC Davis Genome Center (https://github.com/genome/genome/blob/master/lib/perl/Genome/Site/TGI/Hmp/HmpSraProcess/trimBWAstyle.usingBam.pl).

After taxonomic profiling (below), ecologically abnormal WMS samples were identified for further per-sample quality control based on median species-level Bray–Curtis dissimilarity to other samples from the same body site. If the median dissimilarity of a sample exceeded the upper inner fence (1.5 times the interquartile range above the third quartile) for all median dissimilarities from its body site, the sample was labelled an outlier and discarded. This process removed 86 (3.6%) WMS samples that were highly atypical for their respective body sites. Downstream analyses used the remaining 2,355 samples.

### Taxonomic and strain profiling

Taxonomic profiling of the metagenomic samples was performed using MetaPhlAn2^20^, which uses a library of clade-specific markers to provide panmicrobial (bacterial, archaeal, viral, and eukaryotic) profiling (http://huttenhower.sph.harvard.edu/metaphlan2). MetaPhlAn2 profiles recapitulated observed ecological patterns from HMP1 (Extended Data Fig. 1b), and agreed with direct read mapping to reference genomes. Mapped reads covered an average of 81.7% (median 92.8%) of the reference genomic sequence of each modestly dominant strain (comprising at least 5% of the community) across all samples. Mean coverage depth (total base pairs in aligned reads divided by total base pairs in reference genome) for these strains over all samples was 3.9×, with depth-of-coverage means varying widely by body site from 0.04× (right antecubital fossa) to 11.1× (tongue dorsum) (Supplementary Table 8). Batch effects were not visible in the first two axes of variation within each body site (Extended Data Fig. 1d).

Strain characterization was performed using StrainPhlAn^14^. StrainPhlAn characterizes single-nucleotide variants in the MetaPhlAn2 marker genes for an organism. For a given sample, we required a minimum of 80% of markers for a given species to have a minimum mean read depth of 10×, to ensure sufficient data to perform haplotype calling. In total, 151 species satisfied these requirements in at least two WMS samples (Supplementary Table 2). Distances between strains were assessed using the Kimura two-parameter distance^17^ (available from Extended Data Table 1b). Both MetaPhlAn2 and StrainPhlAn were used with their default settings.

Reference genome coverage was scored by the complement of the asymmetric phylogenetic distance (1 − UniFrac G^41^) between HMP1-II strains and reference genomes. All coverage estimates are presented in Supplementary Table 2.

### Niche-association score

Species with niche-associated subspecies clades were detected by a measure similar to the silhouette score, which compares the mean phylogenetic divergence of strains within each body site to the divergence of strains (within the same species) spanning body sites. Specifically, we first define a body site dissimilarity score *D*(*u*, *v*) for a given species at body sites *u* and *v* as:where *S*_*x*_ is the set of samples that pass the StrainPhlAn coverage requirements in body site *x*, and *d*(*i*, *j*) is the Kimura two-parameter distance between dominant haplotypes in samples *i* and *j*. The niche-association score *A* for each species (Fig. 1b) was then defined as the maximum observed *D*(*u*, *v*) over all directed pairs of body sites *u* and *v* where the StrainPhlAn coverage requirements were met for at least five samples in both sites. That is, for a set of body sites *B*:One concern with this score is that greater technical difficulty in single-nucleotide-variant calling in one site may result in apparent niche association where there is none. This is not a concern here, however, as all sites for which the niche-association score was calculated were oral sites with similar technical variability (Fig. 1a). This is a by-product of the limitation that the species were required to have a sufficient presence (five samples passing the StrainPhlAn coverage requirements) at multiple sites, which was not possible outside of the ecologically more similar set of oral sites.

### Functional profiling

Functional profiling was performed using HUMAnN2^24^ (http://huttenhower.sph.harvard.edu/humann2). In brief, for a given sample, HUMAnN2 constructs a sample-specific reference database from the pangenomes of the subset of species detected in the sample by MetaPhlAn2 (pangenomes are precomputed representations of the ORFs of a given species^42^). HUMAnN2 then maps sample reads against this database to quantify gene presence and abundance on a per-species basis. Remaining unmapped reads are further mapped by translated search against a UniRef-based protein sequence catalogue^43^. Finally, for gene families quantified at both the nucleotide and protein levels, HUMAnN2 reconstructs pathways from the functionally characterized subset and assesses community total, species-resolved, and unclassified pathway abundances based on the MetaCyc pathway database^44^.

Analyses of metabolic pathway coreness were focused on 1,087 HMP1-II metagenomes representing the first sequenced visit from each subject at the six targeted body sites. Follow-up samples and technical replicates for a given (subject, body site) combination were excluded to avoid biasing population estimates in their direction. We defined a ‘core’ pathway at a particular body site as one that was detected with relative abundance >10^−4^ in at least 75% of subject-unique samples. We further filtered these highly prevalent pathways to ensure sensible taxonomic range and confident taxonomic attribution. Specifically, a potential core pathway was excluded either if its BioCyc^44^-annotated taxonomic range did not include any human-associated microbial genera (defined as genera detected in at least 5 HMP subjects with relative abundance >10^−3^), or if >50% of pathway copies had ‘unclassified’ taxonomic attribution in >25% of samples. These filtering criteria yielded a total of 950 core (pathway, body site) associations covering 258 unique MetaCyc pathways. Notably, these numbers were reasonably insensitive to the exact parameter settings described above, provided that the overall definition of coreness encompassed (1) a majority population prevalence (that is, >50%), (2) a non-extreme detection threshold [that is, below (number of pathways)^-1^], and (3) some form of taxonomic filtering to limit false positives (for example, to rare variants of otherwise common pathways; Supplementary Table 9).

We quantified the taxonomic range of a pathway as the fraction of unique genera to which it was annotated in BioCyc. We subdivided this measure into ranges over ‘human-associated’ and ‘non-human-associated’ genera (as defined above), and focused on the latter measure to avoid circular reasoning (a function that is broadly distributed across human-associated taxa would be enriched in the human microbiome by definition). As a further control, we also directly applied HUMAnN2 to its underlying pangenome database to associate pathways with >4,000 microbial species. To conservatively define core pathways as ‘enriched to the human microbiome,’ we required them to be annotated to <10% of non-human-associated genera in BioCyc, and also directly annotated to <10% of non-human-associated pangenomes. The second criterion further reduced cases of rare variants of common pathways (as defined by MetaCyc) being called as enriched in metagenomes owing to cross-detection of the common pathway.

We defined a core pathway to be strongly enriched in a particular body site if the first quartile of the abundance of the pathway at that site was >2× larger than the third quartile of abundance at sites from all other body areas (that is, the focal and background abundance distributions must be very well separated, as opposed to just significantly different). Notably, this definition only requires core pathways at oral body sites to separate well from non-oral sites, and not other oral sites (very few core pathways at oral body sites were strongly enriched relative to other oral sites).

We investigated the relationship between coreness and essentiality of functions using a dataset of around 300 essential COG^45^ gene families determined in *E. coli*^46^ (the ‘Keio collection’). We computed COG abundance across the 1,087 metagenomes introduced above by summing the abundance of individual UniRef gene families (as computed by HUMAnN2) according to UniProt-derived COG annotations^47^. We considered a COG to be confidently detected in a sample if its relative abundance exceeded 10^−4^. Among detected COGs, essential COGs (*n* = 272) were both more globally prevalent than non-essential COGs (*n* = 3,629; median 0.94 versus 0.24) and core to more body sites (mean 4.7 versus 1.2; core defined here as >75% prevalent within-site); both trends were highly statistically significant (*P* < 0.001) by Wilcoxon signed-rank tests and robust to a smaller detection threshold (10^−6^).

### Gaussian process dynamics modelling

A Gaussian process is a nonparametric probabilistic model for performing inferences about sampled continuous functions. This section covers the justification of the specific Gaussian process model used to model microbial and functional abundances (referred to here as ‘features’) in the microbiome, and discusses its assumptions, advantages and drawbacks. Implementation details are presented in the following section.

In a Gaussian process, the joint distribution of the modelled function at any finite set of points follows a multivariate normal distribution. Without loss of generality, Gaussian processes can be parameterized solely by their covariance function or kernel, defining the covariance of the output between any pair of sample points. This pairwise definition permits the use of the irregular temporal sampling present in the HMP1-II dataset (Extended Data Fig. 4a). The shape of the covariance function of the Gaussian process determines several properties of the modelled function, such as its smoothness, how quickly it changes, and which features of the input vector it is sensitive to. Our first goal here was thus to assess the strength of the evidence for several common covariance functions describing biologically meaningful behaviours, and to determine which components should be included in a parsimonious model that captures the observable dynamics for the majority of features. The candidate set of covariance functions we considered includes: fast variation (‘biological noise’), inter-individual differences, an Ornstein–Uhlenbeck process, a squared-exponential covariance function, and seasonal dynamics with a period of one year (formulae can be found in Supplementary Table 10).

All candidate covariance functions describe stationary processes, given the inherently limited state space of relative abundances, although they differ in their temporal dynamics and in their implications for the biological systems that generate these behaviours. "Fast variation," meaning variation on a timescale faster than measurable, is represented by a Gaussian white noise process. Inter-individual differences are modelled by constant covariance between samples from the same person. The two time-varying components, the Ornstein–Uhlenbeck process and the squared-exponential covariance function, both describe monotonically decreasing covariance as the difference in time between two samples increases; that is, time points closer to one another will be more similar than those farther apart. These two functions primarily differ in the smoothness of the underlying function. The Ornstein–Uhlenbeck process is the only stationary Markovian Gaussian process with non-trivial covariance over time, and produces functions that are not differentiable, and thus very jagged, resembling Brownian motion. For example, this covariance function is expected for the abundance of a slowly changing feature under continuous stochastic perturbation from the environment. Meanwhile, the squared-exponential covariance function describes functions that are infinitely differentiable, and are thus extremely smooth. This function implies a considerable amount of latent state relevant to the process generating the abundance of the feature. Both of these time-varying covariance functions are parameterized by their length scale, the characteristic time scale at which the function changes. Lastly, the seasonal component is represented by the canonical periodic covariance function from Gaussian process literature, with its period fixed at one year, but with an unknown length scale. Here, a model refers to a combination of these covariance functions.

Models were compared based on their marginal likelihoods (also termed ‘evidence’), reported in bits (that is, log_2_ ratio of marginal likelihoods = log_2_ Bayes factors) of evidence against a given model when compared to the best model for a feature (Supplementary Table 10). More than 3.3 bits is considered strong evidence against a model, and more than 6.6 bits is considered decisive. Marginal likelihoods were estimated from Markov chain Monte Carlo (MCMC) samples of the posterior distribution by a truncated harmonic mean of the un-normalized posterior distribution at the sampled points. Truncation was performed, as this estimator is known to have poor convergence characteristics because MCMC samples with very low likelihoods have an unreasonable influence on the harmonic mean. Comparisons were performed for models fit to the abundances of the top 10 most prevalent species (with at least 70% non-zero abundances) and top 5 most abundant pathways at each targeted body site (Supplementary Table 10). Comparisons were also performed for a set of simulated features with known dynamics (‘controls’), which were sampled from the corresponding Gaussian process with 5% of variance due to technical noise and the remaining variance distributed evenly between components.

To determine which of these components have statistical support in the data, we employed a standard greedy search through the space of possible models, which starts from the simplest model (all variation is technical) and iteratively rejects simpler models in favour of a more complex one if the evidence against the simpler model exceeded six bits. The set of more complex models considered at each iteration are those with only one more parameter, and contain the simpler model as a special case (pseudocode presented in Supplementary Table 6). This procedure selected models that included the two simplest components, biological noise and inter-individual differences, 47 and 53 times among the 72 features tested, respectively. Among more complex components, the Ornstein–Uhlenbeck component was selected 13 times, whereas neither the squared-exponential covariance function nor the seasonal component were selected for a single tested feature. These trends were robust to increases in the model rejection threshold, with the evidence for the Ornstein–Uhlenbeck component remaining significant to at least 10 bits, whereas the squared-exponential covariance function and seasonal components are only selected for more lenient thresholds (≤4 bits). We note, however, that this procedure had difficulty identifying the squared-exponential covariance function and seasonal components in control samples that included other components (in particular, biological noise), indicating that these components are difficult to distinguish given the available temporal sampling pattern. Thus, although the data clearly currently prefer the Ornstein–Uhlenbeck component over the squared-exponential covariance function, and do not support the inclusion of a seasonal component, we are not sufficiently powered to eliminate these as potentially significant contributors to the dynamics of the microbiome. Finally, the null model with only technical noise was rejected for 71 out of the 73 features, often with very high evidence (median 69.6 bits).

For the remainder of the analysis, we thus converged on a model with four components: inter-individual differences, an Ornstein–Uhlenbeck process, biological noise, and technical noise. Let *U*, *T*, *B*, and *N* be the respective magnitudes of these components, and *l* be the timescale of the Ornstein–Uhlenbeck process. Estimation of these parameters (hyperparameters in Gaussian process nomenclature) was performed by fitting a Gaussian process with the following covariance function to all features (species and pathways) with at least 75% prevalence within a site (Fig. 3, Supplementary Table 5):This function describes the covariance between samples i and j, where *t*_*x*_ and *s*_*x*_ are respectively the sampling date and subject identifier of sample *x*. All four parameters were fit simultaneously by MCMC (next section). Since the three magnitude components must sum to the variability of the population, this can be seen as a decomposition of variance into sources of variability that differ in their temporal signature. As we are interested only in the three biological components here, we therefore normalize out the estimated technical noise component (that is, [*U*, *T*, *B*] *N*) before visualizing the decomposition on a standard ternary plot (Fig. 3b, c). For illustration, we show three examples that illustrate the three types of dynamics on a plot designed to allow a direct comparison between the data and the fit Gaussian processes (Extended Data Fig. 4d–f).

The identifiability of any component of a time-dependent model is limited by the temporal sampling pattern available. The current dataset contains only up to three time points per person, with the time between samples roughly evenly distributed between one month and one year for each body site (Extended Data Fig. 4a). Processes too fast to measure will contribute to the biological noise component, whereas processes much slower than the maximum time intervals available contribute to the inter-individual component. We tested what time scales would be detected by the Ornstein–Uhlenbeck component, and which would contribute to the inter-individual or biological noise components, by simulating data from Ornstein–Uhlenbeck processes of varying length scales and performing parameter fits (Extended Data Fig. 4b). These show that the time-varying component is sensitive to processes with characteristic length scales of around 3 to 24 months.

We note that the resolution of the time-varying component is only possible because of the large spread in the time differences between samples available in the HMP1-II dataset (Extended Data Fig. 4a). In another common longitudinal study design, in which a small number of samples are gathered per person with a fixed time interval between them, this would not be possible, although this design may make the analysis simpler (samples can be grouped by time point and a method such as Gaussian processes would not be necessary). Likewise, richer longitudinal data in the form of longer time series would allow even more to be inferred about the dynamics of the microbiome. Of particular interest, this would enable differences in the temporal component(s) to be resolved between people. Here, with only up to three time points per person, the fit model parameters describing temporal changes (*B*, *T*, and *l*) are only a best-fit over the population. Such a sampling pattern would also provide the opportunity to differentiate more conclusively between the Markovian Ornstein–Uhlenbeck process and other possible non-Markovian processes (such as described by the squared-exponential covariance function, or an intermediate such as the Matérn covariance functions), indicative of latent state or time-delayed events in the microbiome.

The HMP1-II dataset also includes many technical replicates (252 in total), which were instrumental in distinguishing the two fast-varying components (biological and technical noise). We encourage the addition of a non-trivial number of technical replicates in future longitudinal studies, not simply for validation but also to allow a quantitative characterization of diversity that is not captured in the remainder of the experiment owing to limited sampling rates. Since technical noise is also estimated with the other variance components, estimates of the relative magnitude of the technical noise are also reported (Supplementary Table 5). The proportion of variance due to technical noise was generally lower for species abundances (median of 5.4%, 90th percentile of 19.3%) than for pathways (median of 16%, 90th percentile of 44%), consistent with the observation that true biological variation between pathway abundances is lower than between species abundances^1^. Noise levels in pathways were predominantly influenced by body site, with pathways in the anterior nares having the greatest noise (median of 40%).

We assessed the accuracy of the parameter fitting process under these noise conditions by simulating samples from mixtures of the three components and performing parameter fits for each targeted body site (Extended Data Fig. 4c). For all noise levels, pure components were always inferred with high confidence, with inter-individual differences being the most identifiable. Mixtures of inter-individual dynamics with biological noise were also confidently recovered, whereas mixtures of inter-individual and biological noise were more variable, and mixtures of inter-individual and time-varying dynamics were biased towards a greater influence of time-varying dynamics. Thus, when the time-varying component is present, parameter estimates should be considered biased away from the inter-individual corner of the ternary diagram. Mixtures of all three components had the greatest uncertainty. Among body sites, inferences at the anterior nares and posterior fornix sampling distributions were the most unreliable, owing to the relatively limited number of samples at these sites (Extended Data Fig. 4a), reflected as a large number of highly uncertain features at these sites (Fig. 3). At 20% technical noise (the 90th percentile of the noise distribution for species), parameter estimates degrade noticeably, and tend towards the mean of the prior (an even mixture of all components). This therefore results in the low-confidence species and pathways tending to locate towards the centre of the ternary diagrams (Fig. 3).

We note that for a particular feature (microbe or pathway abundance), each of the non-technical components represents the sum of all processes with that temporal signature that affect that feature, and these do not necessarily reflect intrinsic properties of the feature. Examples of extrinsic processes that are likely to produce biological noise include, among others, day-to-day dietary differences, the timing of sample collection relative to meals, tooth brushing and other personal hygiene, spatial variation of the microbiome within subjects (for example, gradients across the stool), and weekend/workday differences. Extrinsic sources of inter-individual differences may arise from culture/ethnicity (ethnicity is strongly associated with the abundances of several microbes^1^), differences in habits (for example, habitual versus infrequent tooth brushers and flossers), and long-term dietary differences, among others. Finally, time-varying processes may include properties such as weight or slowly changing preferences in diet.

### Gaussian process parameter optimization details

All parameter fits and model comparisons were performed by MCMC sampling with the GPstuff toolbox version 4.6 in MATLAB. Before fitting, relative abundances were first arcsine square-root transformed, filtered for outliers using the Grubbs outlier test (significance threshold 0.05), and standardized to have zero mean and unit variance. A gamma-distributed prior with shape 3.1 and mean 10 months was imposed on the lengthscale parameter of all time-varying components. These parameters for *l* were selected based on the intervals between samples, and guarantee that the model is identifiable when the biological noise and/or inter-individual difference components are included by ensuring that *l* is neither too short nor too long. All parameters of all models were fit simultaneously. All models were fit using a Gaussian likelihood. This function performs poorly for highly non-Gaussian distributions, which frequently occur in microbiome data in the form of zero-inflated abundance distributions. For this reason, the dynamics analysis was performed for highly prevalent features (species with ≥ 75% prevalence within a site, and core pathways). One exception was made for this: species with mean abundance when present at ≥ 2% and non-zero in at least 50 samples were also included, so as to include important species such as *Prevotella copri* that have lower prevalence but exceptional abundance when present. Other models specifically accounting for zero-inflation (both technical and real) will be needed to study the dynamics of the rarer microbiome.

Evidence presented in Supplementary Table 5 was calculated from 5 MCMC chains per model, with 150 samples after a 20 sample burn-in, which were started from a random point in the prior distribution. Parameter estimates presented in Fig. 3 and Supplementary Table 5 were fit with the additional constraint that *U* + *T* + *B* + *N* = 1, to eliminate an additional degree of freedom from the model. A Dirichlet(1, 1, 1, 1) prior was imposed on [*U*, *T*, *B*, *N*]. For each feature tested here, a more thorough MCMC sampling was performed than for the model selection, consisting of 10 chains with 200 samples each (after 30 burn-in and thinning every other sample), starting from a random point from the prior distribution. In all cases, all parameters were fit simultaneously. Convergence was assessed with the  statistic^48^. Over all 196 species and 950 pathways tested, 97% of  statistics were <1.1 for all parameters (median 1.01, max 1.17), indicating good convergence.

### Association testing between microbiome features and phenotypic covariates

Associations between microbial and pathway abundances and metadata were determined using MaAsLin^1,49^. MaAsLin tests a sparse multivariate generalized linear model against each feature independently. Relative abundances were first arcsine square-root transformed for variance stabilization, and the Grubbs test was used (significance level 0.05) to remove outliers. A univariate prescreen was applied using boosting to identify potentially associated features, and significantly associated covariates among the remaining features were identified with a multivariate linear model without zero-inflation. Unless otherwise stated, a final FDR < 0.1 (Benjamini–Hochberg controlled across feature tests) was used as a significance threshold.

The same model was applied to all features (microbial and pathway) during this analysis and included the following covariates: broad dietary characterization, whether the subject was breastfed, temperature, introitus pH, posterior fornix pH, gender, age, ethnicity, study day processed, sequencing centre, clinical centre, number of quality bases, percentage of human reads, systolic blood pressure, diastolic blood pressure, pulse, whether the subject had given birth, HMP1/HMP1-II, and BMI. A summary of these metadata can be found in Extended Data Table 1a. Of note, several recently identified confounders such as transit time^8^ for stool samples were not collected during sampling.

### Benchmarking and assembly protocol design

We benchmarked several assemblers including IDBA-UD^38^, MetaVelvet^50^, SOAPDenovo2^51^, Newbler (Roche, Basel, Switzerland), Ray^52^, SPAdes^53^, and Velvet^54^ using eight samples (SRS017820, SRS014126, SRS052668, SRS017820, SRS048870, SRS020220, SRS057205 and SRS017820) across five body sites that represented a range of metagenomic complexity. On the basis of the assembly size, median length, fragmentation level, and N50 length, we chose IDBA-UD to process all HMP1-II samples.

### Digital normalization

Following quality control, sequence reads for each sample were run through a ‘digital normalization’ pipeline before assembly. This process was designed to reduce, as much as possible, the volume of information from the most dominant source taxa (without sacrificing the ability to assemble what remains) so that lower-abundance taxa could be assembled more evenly, instead of having their reads discarded by the assembler software as not being sufficiently covered (compared to the dominant taxa).

Median *k*-mer coverage was first estimated for all reads using the khmer Python library^55^. These data were then used to filter input reads so as to normalize *k*-mer coverage within preselected bounds: for each *k*-mer of length 20 nucleotides in each read, the total number of observations of the *k*-mer was used as a proxy for coverage. Reads for which median *k*-mer coverage was already greater than 20 were discarded. Remaining reads were then trimmed at the first instance of a single-copy *k*-mer (representing putative error sequences). Reads with a post-trim length of less than the *k*-mer length (20 nucleotides) were also discarded. Surviving reads were trimmed again, this time at the first instance of a high-abundance (>50×) *k*-mer; again, reads whose post-trim length was less than 20 nucleotides were discarded. For remaining reads, we re-normalized (based on median *k*-mer coverage as in the first step) to remove all reads whose median *k*-mer coverage was >5×. This is a more aggressive filter on putatively redundant sequences, after elimination of initial reads with highly-overrepresented (redundant) *k*-mers or severely under-represented (error) *k*-mers.

For subsequent assembly after this quality control and normalization, we increased *k* to 32 nucleotides (to maximize sensitivity on the remaining reads) and built an overlap graph from all remaining reads. This graph was then partitioned into groups of reads with a high likelihood of internal overlap, separating components at precomputed ‘stoptags’: *k*-mer sequences automatically identified by khmer in its initial profiling scan as unreliable assembly-traversal nodes. Reads were then extracted from each such partition into separate FASTA files. Each partition was tested for more coherent subgroups, beginning with the least consistent (ranked in order of graph separability). Re-partitioning was carried out as above, but with more aggressive parameters: stoptags in the initially-computed overlap graph were explicitly detected and removed before re-partitioning (which included the generation of new stoptags from the remainder of the graph after removal of the earlier ones). The least-consistent read group was broken into sub-partitions exactly once in this way: further iteration risks overfitting and is not guaranteed to converge to a meaningful result.

### IDBA-UD assembly and post-processing

Following digital normalization, each final partition was assembled independently of the others with IDBA-UD. For values of *k* in (20, 30, 40,..., 80), IDBA-UD will attempt to assemble its partition (via de Bruijn graph methods) using *k*-mers of size *k*, and will then merge and extend results from all passes to produce a final assembly of that partition (requiring a minimum contig length of 100 nucleotides). For each sample (or pool), all (independently produced) partition assemblies were then concatenated. As a final step to reduce any redundancy present in the final concatenated assembly, we merged and extended all assembled contigs (across all partitions), based on overlaps of 40 nucleotides or more, to produce a final ‘consolidated’ sequence collection.

### Quality assessment

To assess assembly quality we undertook a number of post-assembly quality control checks, including an examination of the rate at which reads aligned to assemblies as well as identifying chimaeras, which are a potential problem caused by mis-assemblies.

To check for what portion of the reads were incorporated into the assembly, sample reads were aligned against their assembly using Bowtie v1, resulting in counts for reads with at least one alignment and for those that failed to align. Total reads include reads from the human host. Because human reads were masked as all Ns by SRA using BMTagger, the human reads would affect the portion of unaligned reads. To assess the effect, we counted the number of masked reads to obtain a count for human reads. These are summarized by body site in Extended Data Fig. 7c.

### Assembly protocol validation

To examine the rates of chimaeric contigs and mis-assemblies, we undertook an assembly assessment of 2 mock datasets generated during the HMP, one in which the community was created with all 21 organisms in equal abundance (‘even’), and one with staggered abundances. We assembled these mock communities using the same protocol and aligned assembled contigs for both sets against all 21 input genomes. We found that 94.21% and 96.84%, respectively, of all assembled contigs aligned uniquely to a single reference genome for the even- and staggered-coverage mock communities (‘aligned’ here means aligned with ≥ 95% sequence identity over ≥ 95% of their length). Contigs aligned to closely related *Staphylococcus* and *Streptococcus* strains exhibited slightly more non-exclusive matching (or cross-matching) than contigs aligned to other strains. For the even set, an average of 97.85% of all *Staphylococcus*- and *Streptococcus*-aligned contigs were uniquely aligned to their reference strain, with an average of 92.98% for the staggered set, as compared to averages across all other strains of 99.89% (even) and 98.98% (staggered), neatly reflecting the inherent genetic ambiguity of these taxonomically narrow subgroups yet showing a very strong ability to distinguish between related strains.

Recovery statistics do not correlate well with input coverage in the staggered set, implying that our pipeline (given a minimum of 4× coverage) is robust against differences in relative abundance of up to three orders of magnitude at these scales. Phylogenetic proximity, in this case, seems to show a greater influence on uniqueness of assembly (albeit still a very weak one) than does coverage. Fractions of contigs not aligning to any of the 21 reference strains (over ≥ 95% of their length at ≥ 95% identity) were 5.6% and 3.0% for the even and staggered sets respectively; we can thus postulate these proportions to be upper bounds on the combined rates of chimaeras and mis-assemblies produced by our pipeline, consistent with other chimaera assembly metrics^56^.

### Annotation

Detection of ORFs within assembled contigs was performed using Metagenemark-3.25^57^. The resulting ORF sequences were used as input for searches against (1) UniRef100^58^ using RAPSearch2^59^; (2) Pfam^60^ and TIGRfam^61^ HMM models using hmmer-3.0^62^; (3) TMHMM^63^ for the identification of transmembrane helices; and (4) a regular expression search for membrane lipoprotein lipid attachment sites for the identification of putative signal peptides. The latter three searches were run as implemented in the Ergatis workflow monitoring system^64^.

Annotation was assigned by Attributor (https://github.com/jorvis/Attributor) using a hierarchical scheme developed out of the IGS Prokaryotic Annotation Pipeline^65^. Attributor assigns common names, gene symbols, enzyme commission (EC) numbers and Gene Ontology (GO) terms, as applicable, based on a hierarchy of evidence including hits to HMM models, UniRef100 sequences, TMHMM predicted helical spans, and lipoprotein motifs. Assignments are exclusive, meaning that for each ORF, Attributor takes the strongest piece of evidence available and assigns all attributes possible based on that evidence. Attributes are not assigned from multiple sources to ensure that annotation attributes assigned to a single ORF do not conflict. Attributor annotation was output as gff3 and FASTA files (Extended Data Table 1b).

### Rarefaction curves

Rarefaction curves were generated by extracting predicted polypeptides from the MetaGeneMark output for each sample, and estimating a ‘unique gene family’ count for rarefied sample size *n* as follows, using usearch v.8.1.1861 x64^66^: (1) Concatenate the MetaGeneMark predicted polypeptides from a random sampling of *n* samples that were not technical replicates, eliminating duplicates; (2) Sort sequences by decreasing length; (3) Cluster sequences at 90% identity (using usearch cluster_fast); (4) Retrieve the ‘unique gene family’ count from the results. The number of unique clusters was estimated from 50 random subsets for each *n*. This procedure was repeated for each body site for *n* = 1,10,20,... until the number of unique samples available at the body site.

### Mapping reads to reference genomes

In addition to taxonomic and functional profiling as above, the individual raw reads of all samples were aligned directly to MetaRef^42^ reference genomes. Before alignment, all reads with 80% or higher percentage of Ns were discarded using the Biocode fastq::filter_fastq_by_N_content utility (https://github.com/jorvis/biocode/blob/master/fastq/filter_fastq_by_N_content.py). Bowtie2^67^ (v2.2.4) was then used to align reads to reference genomes using the default, paired-end alignment options and including the singleton reads. The resulting SAM files were converted to BAM, sorted, and then partitioned into two separate files per sample— one of only matching reads and the other of unaligned reads. This entire pipeline is encapsulated in the Biocode generate_read_to_metaref_seed_alignment.py pipeline script (https://github.com/jorvis/biocode/blob/master/sandbox/jorvis/generate_read_to_metaref_seed_alignment.py).

### Mapping reads to assembled contigs

The quality-trimmed reads from each sample were mapped back onto the assembled contigs from that same sample using Bowtie (v0.12.9) with a 512 MB max best-first search frames value, Phred33 score quality setting, 21 base-pair seed length, and limit of 2 mismatches per seed. All alignments per read were reported (unless there were more than 20 for a given read) with hits guaranteed best stratum and ties broken by quality. Hits in sub-optimal strata were not reported.

### Code availability

Code for the annotation pipeline and the Gaussian Process analysis are available from Extended Data Table 1b.

### Data availability

Sequence data are available from the HMP DACC (http://hmpdacc.org) or on Amazon (https://aws.amazon.com/datasets/human-microbiome-project/); WMS reads and accompanying metadata are available at the Sequence Read Archive (SRA; https://www.ncbi.nlm.nih.gov/sra) and the Database of Genotypes and Phenotypes (dbGaP; https://www.ncbi.nlm.nih.gov/gap) under two studies: SRP002163 (BioProject PRJNA48479), and SRP056641 (BioProject PRJNA275349). Public and private metadata from Extended Data Table 1 are available with the metagenomic taxon abundances table from the HMP DACC (https://www.hmpdacc.org/hmsmcp2/), and through the dbGaP with accession number phs000228.v3.p1, respectively. All other data are available from the corresponding author upon reasonable request.

## Supplementary information

Supplementary Information This file contains a Supplementary Discussion and full legends for Supplementary Tables 1-11. (PDF 217 kb)

Reporting Summary (PDF 67 kb)

Supplementary Tables This file contains Supplementary Tables 1-11. (XLSX 703 kb)

## Figures and Tables

**Extended Data Table 1:**
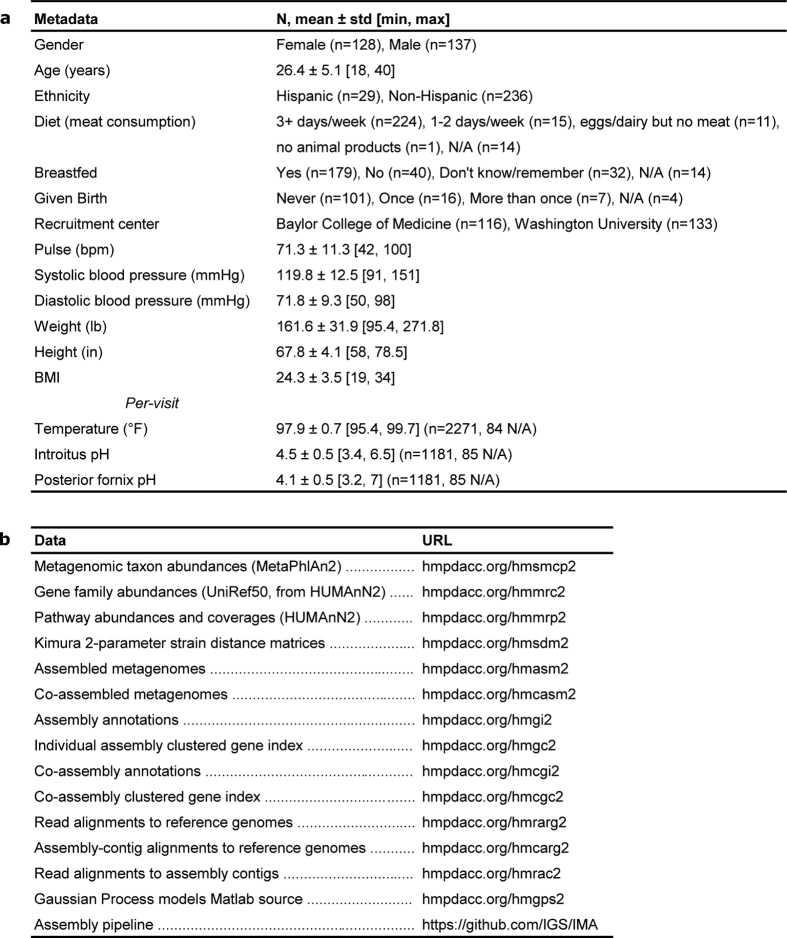
Metadata summary and data availability

**a**, Overview of the characteristics of the 265 subjects for which shotgun metagenomes are available in the HMP1-II dataset. Group sizes are given for categorical data, mean ± s.d. and [minimum, maximum] are given for continuous data. Subjects with recent antibiotic or probiotic usage were specifically excluded from the study^40^. Transit time, an important recently identified confounder in gut microbiome studies^68^, was not collected as part of the sampling metadata. Metadata availability: public metadata are available with the metagenomic taxon abundances table (https://www.hmpdacc.org/hmsmcp2/); private metadata are available through dbGaP with accession number phs000228.v3.p1. **b**, All data products are available at the HMP DACC at the URLs given in the table. Data can be downloaded using standard protocols such as http, ftp, and the Aspera protocol.

## Data Availability

BioProject
PRJNA275349
PRJNA48479
Sequence Read Archive
SRP002163
SRP056641

## PowerPoint slides

PowerPoint slide for Fig. 1
PowerPoint slide for Fig. 2
PowerPoint slide for Fig. 3
PowerPoint slide for Fig. 4

## References

[1] The Human Microbiome Project Consortium. Structure, function and diversity of the healthy human microbiome. *Nature* **486**, 207–214 (2012) 10.1038/nature11234 PMC3564958 22699609

[2] Lloyd-Price J, Abu-Ali G, Huttenhower C (2016). The healthy human microbiome. Genome Med..

[3] Gensollen T, Iyer SS, Kasper D L, Blumberg RS (2016). How colonization by microbiota in early life shapes the immune system. Science.

[4] Honda K, Littman DR (2016). The microbiota in adaptive immune homeostasis and disease. Nature.

[5] Qin J (2010). A human gut microbial gene catalogue established by metagenomic sequencing. Nature.

[6] Li J (2014). An integrated catalog of reference genes in the human gut microbiome. Nat. Biotechnol..

[7] Beaumont M (2016). Heritable components of the human fecal microbiome are associated with visceral fat. Genome Biol..

[8] Falony G (2016). Population-level analysis of gut microbiome variation. Science.

[9] Zhernakova A (2016). Population-based metagenomics analysis reveals markers for gut microbiome composition and diversity. Science.

[10] Si J, You H J, Yu J, Sung J, Ko G (2017). *Prevotella* as a hub for vaginal microbiota under the influence of host genetics and their association with obesity. Cell Host Microbe.

[11] Gonzalez, A. et al. Migraines are correlated with higher levels of nitrate-, nitrite-, and nitric oxide-reducing oral microbes in the American Gut Project cohort. *mSystems*10.1128/mSystems.00105-16 (2016) 10.1128/mSystems.00105-16 PMC5080405 27822557

[12] Oh J (2014). Biogeography and individuality shape function in the human skin metagenome. Nature.

[13] The Human Microbiome Project Consortium. A framework for human microbiome research. *Nature* **486**, 215–221 (2012) 10.1038/nature11209 PMC3377744 22699610

[14] Truong DT, Tett A, Pasolli E, Huttenhower C, Segata N (2017). Microbial strain-level population structure and genetic diversity from metagenomes. Genome Res..

[15] Schloissnig S (2013). Genomic variation landscape of the human gut microbiome. Nature.

[16] Luo C (2015). ConStrains identifies microbial strains in metagenomic datasets. Nat. Biotechnol..

[17] Kimura M (1980). A simple method for estimating evolutionary rates of base substitutions through comparative studies of nucleotide sequences. J. Mol. Evol..

[18] Franzosa EA (2015). Identifying personal microbiomes using metagenomic codes. Proc. Natl Acad. Sci. USA.

[19] Leinonen R, Sugawara H, Shumway M (2011). The sequence read archive. Nucleic Acids Res..

[20] Truong DT (2015). MetaPhlAn2 for enhanced metagenomic taxonomic profiling. Nat. Methods.

[21] Hoffmann C (2013). Archaea and fungi of the human gut microbiome: correlations with diet and bacterial residents. PLoS ONE.

[22] Schwiertz A (2010). Microbiota and SCFA in lean and overweight healthy subjects. Obesity.

[23] Pride DT (2012). Evidence of a robust resident bacteriophage population revealed through analysis of the human salivary virome. ISME J..

[24] Abubucker S (2012). Metabolic reconstruction for metagenomic data and its application to the human microbiome. PLoS Comput. Biol..

[25] Caspi R (2014). The MetaCyc database of metabolic pathways and enzymes and the BioCyc collection of pathway/genome databases. Nucleic Acids Res..

[26] Leonardi R, Zhang YM, Rock C O, Jackowski S (2005). Coenzyme A: back in action. Prog. Lipid Res..

[27] Khakh BS, Burnstock G (2009). The double life of ATP. Sci. Am..

[28] Morkbak AL, Poulsen SS, Nexo E (2007). Haptocorrin in humans. Clin. Chem. Lab. Med..

[29] Roy CC, Kien CL, Bouthillier L, Levy E (2006). Short-chain fatty acids: ready for prime time?. Nutr. Clin. Pract..

[30] Schreiber F (2010). Denitrification in human dental plaque. BMC Biol..

[31] Flint HJ, Scott KP, Duncan S H, Louis P, Forano E (2012). Microbial degradation of complex carbohydrates in the gut. Gut Microbes.

[32] Faith JJ (2013). The long-term stability of the human gut microbiota. Science.

[33] Flores GE (2014). Temporal variability is a personalized feature of the human microbiome. Genome Biol..

[34] Ding T, Schloss PD (2014). Dynamics and associations of microbial community types across the human body. Nature.

[35] Turnbaugh PJ (2007). The human microbiome project. Nature.

[36] Shafquat A, Joice R, Simmons S L, Huttenhower C (2014). Functional and phylogenetic assembly of microbial communities in the human microbiome. Trends Microbiol..

[37] Gajer P (2012). Temporal dynamics of the human vaginal microbiota. Sci. Transl. Med..

[38] Peng Y, Leung HC, Yiu S M, Chin FY (2012). IDBA-UD: a *de novo* assembler for single-cell and metagenomic sequencing data with highly uneven depth. Bioinformatics.

[39] Finn RD (2016). The Pfam protein families database: towards a more sustainable future. Nucleic Acids Res..

[40] Aagaard K (2013). The Human Microbiome Project strategy for comprehensive sampling of the human microbiome and why it matters. FASEB J..

[41] Caporaso JG (2010). QIIME allows analysis of high-throughput community sequencing data. Nat. Methods.

[42] Huang K (2014). MetaRef: a pan-genomic database for comparative and community microbial genomics. Nucleic Acids Res..

[43] Suzek BE, Wang Y, Huang H, McGarvey PB, Wu CH (2015). UniRef clusters: a comprehensive and scalable alternative for improving sequence similarity searches. Bioinformatics.

[44] Caspi R (2016). The MetaCyc database of metabolic pathways and enzymes and the BioCyc collection of pathway/genome databases. Nucleic Acids Res..

[45] Galperin MY, Makarova KS, Wolf YI, Koonin EV (2015). Expanded microbial genome coverage and improved protein family annotation in the COG database. Nucleic Acids Res..

[46] Baba T (2006). Construction of *Escherichia coli* K-12 in-frame, single-gene knockout mutants: the Keio collection. Mol. Syst. Biol..

[47] The UniProt Consortium. UniProt: the universal protein knowledgebase. *Nucleic Acids Res.* **45**, D158–D169 (2017) 10.1093/nar/gkw1099 PMC5210571 27899622

[48] Gelman A, Rubin DB (1992). Inference from iterative simulation using multiple sequences. Stat. Sci..

[49] Morgan XC (2012). Dysfunction of the intestinal microbiome in inflammatory bowel disease and treatment. Genome Biol..

[50] Namiki T, Hachiya T, Tanaka H, Sakakibara Y (2012). MetaVelvet: an extension of Velvet assembler to *de novo* metagenome assembly from short sequence reads. Nucleic Acids Res..

[51] Luo R (2015). SOAPdenovo2: an empirically improved memory-efficient short-read *de novo* assembler. GigaScience.

[52] Boisvert S, Raymond F, Godzaridis E, Laviolette F, Corbeil J (2012). Ray Meta: scalable *de novo* metagenome assembly and profiling. Genome Biol..

[53] Bankevich A (2012). SPAdes: a new genome assembly algorithm and its applications to single-cell sequencing. J. Comput. Biol..

[54] Zerbino DR, Birney E (2008). Velvet: algorithms for *de novo* short read assembly using de Bruijn graphs. Genome Res..

[55] Pell J (2012). Scaling metagenome sequence assembly with probabilistic de Bruijn graphs. Proc. Natl Acad. Sci. USA.

[56] Mende DR (2012). Assessment of metagenomic assembly using simulated next generation sequencing data. PLoS ONE.

[57] Zhu W, Lomsadze A, Borodovsky M (2010). *Ab initio* gene identification in metagenomic sequences. Nucleic Acids Res..

[58] Suzek BE, Huang H, McGarvey P, Mazumder R, Wu CH (2007). UniRef: comprehensive and non-redundant UniProt reference clusters. Bioinformatics.

[59] Zhao Y, Tang H, Ye Y (2012). RAPSearch2: a fast and memory-efficient protein similarity search tool for next-generation sequencing data. Bioinformatics.

[60] Finn RD (2014). Pfam: the protein families database. Nucleic Acids Res..

[61] Haft DH (2013). TIGRFAMs and Genome Properties in 2013. Nucleic Acids Res..

[62] Eddy SR (2011). Accelerated profile HMM searches. PLoS Comput. Biol..

[63] Sonnhammer EL, von Heijne G, Krogh A (1998). A hidden Markov model for predicting transmembrane helices in protein sequences. Proc. Int. Conf. Intell. Syst. Mol. Biol..

[64] Orvis J (2010). Ergatis: a web interface and scalable software system for bioinformatics workflows. Bioinformatics.

[65] Galens K (2011). The IGS standard operating procedure for automated prokaryotic annotation. Stand. Genomic Sci..

[66] Edgar RC (2010). Search and clustering orders of magnitude faster than BLAST. Bioinformatics.

[67] Langmead B, Salzberg SL (2012). Fast gapped-read alignment with Bowtie 2. Nat. Methods.

[68] Roager HM (2016). Colonic transit time is related to bacterial metabolism and mucosal turnover in the gut. Nat. Microbiol..

