# Supplementary material for: Strains, functions and dynamics in the expanded Human Microbiome Project
Source: Nature. 2017 Sep 20;550(7674):61–6. doi: 10.1038/nature23889 (PMC5831082; doi:10.1038/nature23889)
Supplement: Supplementary Information — This file contains a Supplementary Discussion and full legends for Supplementary Tables 1-11. (PDF 217 kb) [file 41586_2017_BFnature23889_MOESM1_ESM.pdf]

doi:10.1038/nature23889

Supplementary Discussion for "Strains, functions, and dynamics in the expanded Human Microbiome Project"

Updated associations between phenotype and microbiome composition

With a ~2.5x increase in the number of different subjects with WMS samples at each targeted body site, we tested for significant correlations between species abundances, pathways, and covariates that the initial HMP dataset was not powered to detect[1] (**Extended Data Fig. 8**; see Methods for included metadata and confounders). Overall, 151 significant associations were found with species abundances (**Extended Data Fig. 8a**, **Table S11**), and 469 were found with pathways (**Extended Data Fig. 8b**, **Table S11**). Previously-significant associations persist in the new analysis (**Extended Data Fig. 9d-f**).

A new noteworthy association includes a negative relationship between the abundance of Firmicutes and whether the subject had been breastfed as an infant (**Extended Data Fig. 9a**), which has not been seen in previous adult cohorts (e.g. ref. [2]). Interestingly, this is in the opposite direction to recent surveys of the developing infant gut microbiome[3]. Having been breastfed was also linked to the oral clades *Neisseria* (**Extended Data Fig. 9b**), *Rothia* and *Veillonella*, previously identified to be differentially abundant in breastfed infants versus formula-fed infants[4]; these clades overlapped with oral microbes that also differed with age independently of breastfeeding status (e.g. **Extended Data Fig. 9c**).

References

1 The Human Microbiome Project Consortium. Structure, function and diversity of the healthy human microbiome. Nature 486, 207-214, doi:10.1038/nature11234 (2012).

2 Falony, G. et al. Population-level analysis of gut microbiome variation. Science 352, 560-564, doi:10.1126/science.aad3503 (2016).

3 Chu, D. M. et al. Maturation of the infant microbiome community structure and function across multiple body sites and in relation to mode of delivery. Nat Med 23, 314-326, doi:10.1038/nm.4272 (2017).

4 Holgerson, P. L. et al. Oral microbial profile discriminates breast-fed from formula-fed infants. J Pediatr Gastroenterol Nutr 56, 127-136, doi:10.1097/MPG.0b013e31826f2bc6 (2013).

Supplementary Table legends

Supplemental Table S1: Detailed body site sampling statistics. The number of subjects for which a given number of visits (1-3) are available with 16S profiles from HMP1[1] (300 subjects), shotgun metagenomes (expanded in HMP1-II, 265 subjects), and where both data types are available for the same visit, stratified across all sampled body sites. Note that a given subject necessarily has less visits with both data types available than with either single data type.

Supplemental Table S2: Strain-level phylogenetic diversity for all species within and between body sites and subjects. Overview of strain diversity (sequence divergence using Kimura two-parameter distance) for each species, including columns indicating: the number of samples with >80% coverage, length of multiple sequence alignment (MSA) used for haplotyping, number of reference genomes, and statistics on distances between strains (number of strain pairs from which statistics were calculated, and their average and standard deviation) between and within: body sites, three-digit zip codes, person, and time points, average distance between all strains, and mean distance to nearest reference genome.

Supplemental Table S3: Co-occurrence/co-exclusion patterns with non-bacterial microbes. Fisher's exact test was used to search for co-occurrence/co-exclusion between non-bacterial species (in particular Archaea and Viruses) and other members of the microbiome at each targeted body site. Species presence was defined as relative abundance >0.1%, and tests were only performed between species with prevalence in [0.05, 0.95]. To avoid biasing this test towards subjects with more sequenced visits, only the first sequenced visit for each subject was used in this test. p-values were adjusted to q-values by Benjamini-Hochberg correction.

Supplemental Table S4: Identification of core pathways. Abundance quartiles for each detected pathway are shown for each of the targeted body sites, for both total community abundances and abundances of unknown taxonomic origin.

Supplemental Table S5: Gaussian process parameter estimates for species and pathway temporal dynamics. For each feature (species or pathway) at each body site, the table shows the feature's prevalence (fraction of samples with >0 abundance), mean abundance when present, and normalized estimates of the parameters of the Gaussian Process (GP) model of the dynamics. The fit GP's covariance function is given in Methods. Parameters were fit with the restriction that $U + T + B + N = 1$. A Gamma-distributed prior with shape 3.1 and scale 10 months was imposed on l, and a Dirichlet(1, 1, 1, 1) prior was imposed on $[U, T, B, N]$. MCMC samples of the posterior distribution of $[U, T, B, N]$ and l were obtained using the GPstuff package in MATLAB to fit the GP to the relative abundances of each feature after arcsin-sqrt transformation, outlier removal using the Grubbs outlier test (significance threshold 0.05), and standardization. The table contains normalized estimates of the variance decomposition, i.e. posterior means of $[U, T, B, N]$ and l, as well as an estimate of the uncertainty of the inference of the three non-technical components, quantified by the variance of the distribution of $(B+U/2, U\sqrt{3/4})/(1\text{-}N)$ (i.e. the points in the ternary plot). Maximum a posteriori (MAP) estimates are also provided for $[U, T, B, N]$ and l.

Supplemental Table S6: Co-assemblies outperform single-assemblies at all targeted body sites. The average contig count, total assembly size, median contig length, and N50 are shown for single- and co-assemblies for 7 body sites. Improvements over single assemblies are seen for all body sites in the contig count and assembly size. Median contig length and N50 are reduced for some body sites, due to a larger fraction of shorter contigs in the co-assemblies.

Supplemental Table S7: Summary of assembly annotation statistics by body site. The average gene count and gene length are shown for both single and co-assemblies. Large improvements are seen for gene count at all targeted body sites in the co-assemblies.

Supplemental Table S8: Read alignment to an extended MetaRef database. We aligned each sample's individual reads to the MetaRef reference genome collection using Bowtie2 with default parameters, preserving all valid hits per read. On average, 39% of post-QC reads aligned to at least one genome in the collection. For further analyses, in each sample, we only retained organisms whose alignment-based relative abundance estimate was 5% or greater in that sample. 1,426 strains in all (total database 4,280 reference genomes) were hit by reads; of these, 335 passed the 5%-abundance threshold in at least one sample. The table, stratified by body site, gives raw counts of input reads and successfully aligned reads, the number of strains passing the 5% threshold for each site, the mean percentage of qualifying reference genomes covered by aligned reads, and the mean depth of coverage for qualifying strains.

Supplemental Table S9: Defining pathway coreness. Pathway coreness was based on three quantitative measurements across HMP1-II samples (#1-3) and one qualitative property (#4): 1) prevalence of the pathway across unique individuals at a particular body site (rows of plots), 2) the detection threshold for considering a pathway "present" (y-axes), 3) the percent of detected pathway copies attributable to species (x-axes), and 4) whether or not the pathway was explicitly linked to human-associated genera in BioCyc (columns of plots). Counts reflect events of a particular pathway being core to a particular site under the given parameters. The definition of coreness becomes more stringent moving from left to right and bottom to top. Our (fairly stringent) definition is highlighted. Notably, counts do not vary dramatically across "reasonable" definitions of coreness, with such definitions including 1) majority (>50%) prevalence; 2) a detection threshold below 1/(the total number of pathways); and 3) some form of taxonomic filtering (either a non-trivial fraction of copies assigned to known species or explicit taxonomic limitation based on pathway annotation).

Supplemental Table S10: New data supports a dynamical model with 3 biological components of variation. Columns C-Z show the evidence ($\log_2$ marginal likelihoods) for a suite of models (combinations of covariance functions) for the top 10 most prevalent species at each site (minimum prevalence 75%) and top 5 most abundant pathways at each site, as well as a set of simulated samples with known dynamics ("controls"), sampled with the temporal sampling pattern in stool. Evidence is presented in terms of the difference in $\log_2$ marginal likelihood between the given model and the optimal model (with greatest marginal likelihood). Coloring is by the strength of the evidence against the given model (red models are very unlikely, while green models cannot be distinguished). Columns AB-AF show the "best" model, as selected by a greedy search (pseudocode presented on the right), with varying model rejection thresholds. The summary table in columns AI-AN summarizes the number of times each component was selected in the "best" set of models for each model rejection threshold.

Supplemental Table S11: Significant associations between species abundances, pathway functional profiles, and host phenotypes. List of all significant associations (FDR<0.1) from multivariate association testing (MaAsLin) for the 6 targeted body sites. Species abundances from MetaPhlAn2 and pathway abundances from HUMAnN2 are both included.
