# Supplementary material for: Strains, functions and dynamics in the expanded Human Microbiome Project
Source: Nature. 2017 Sep 20;550(7674):61–6. doi: 10.1038/nature23889 (PMC5831082; doi:10.1038/nature23889)
Supplement: Supplementary file 5 — PowerPoint slide for Fig. 2 [file 41586_2017_BFnature23889_MOESM5_ESM.ppt]

## Slide 1
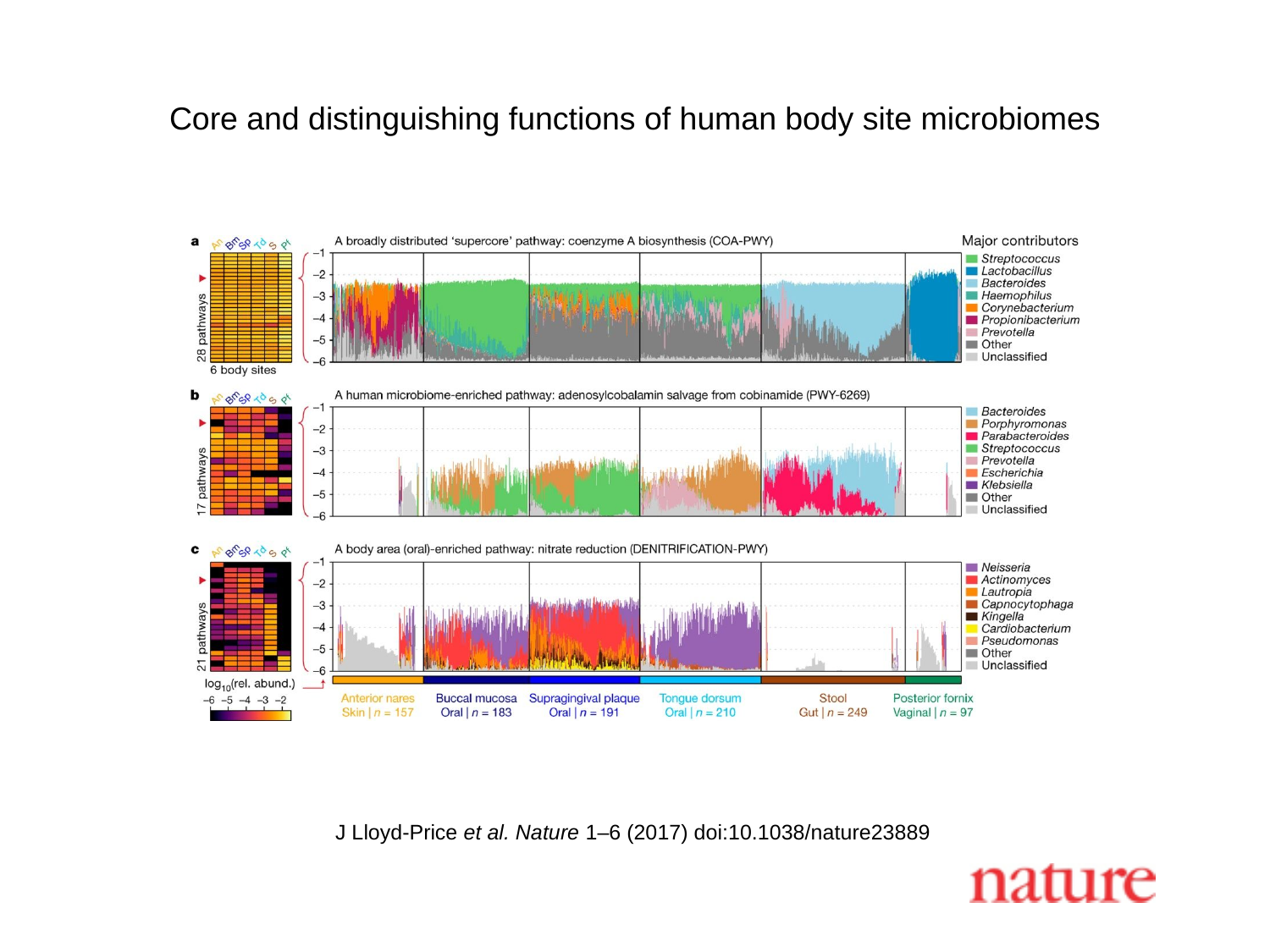

# Core and distinguishing functions of human body site microbiomes
J Lloyd-Price et al. Nature 1–6 (2017) doi:10.1038/nature23889
