# Supplementary material for: Strains, functions and dynamics in the expanded Human Microbiome Project
Source: Nature. 2017 Sep 20;550(7674):61–6. doi: 10.1038/nature23889 (PMC5831082; doi:10.1038/nature23889)
Supplement: Supplementary file 6 — PowerPoint slide for Fig. 3 [file 41586_2017_BFnature23889_MOESM6_ESM.ppt]

## Slide 1
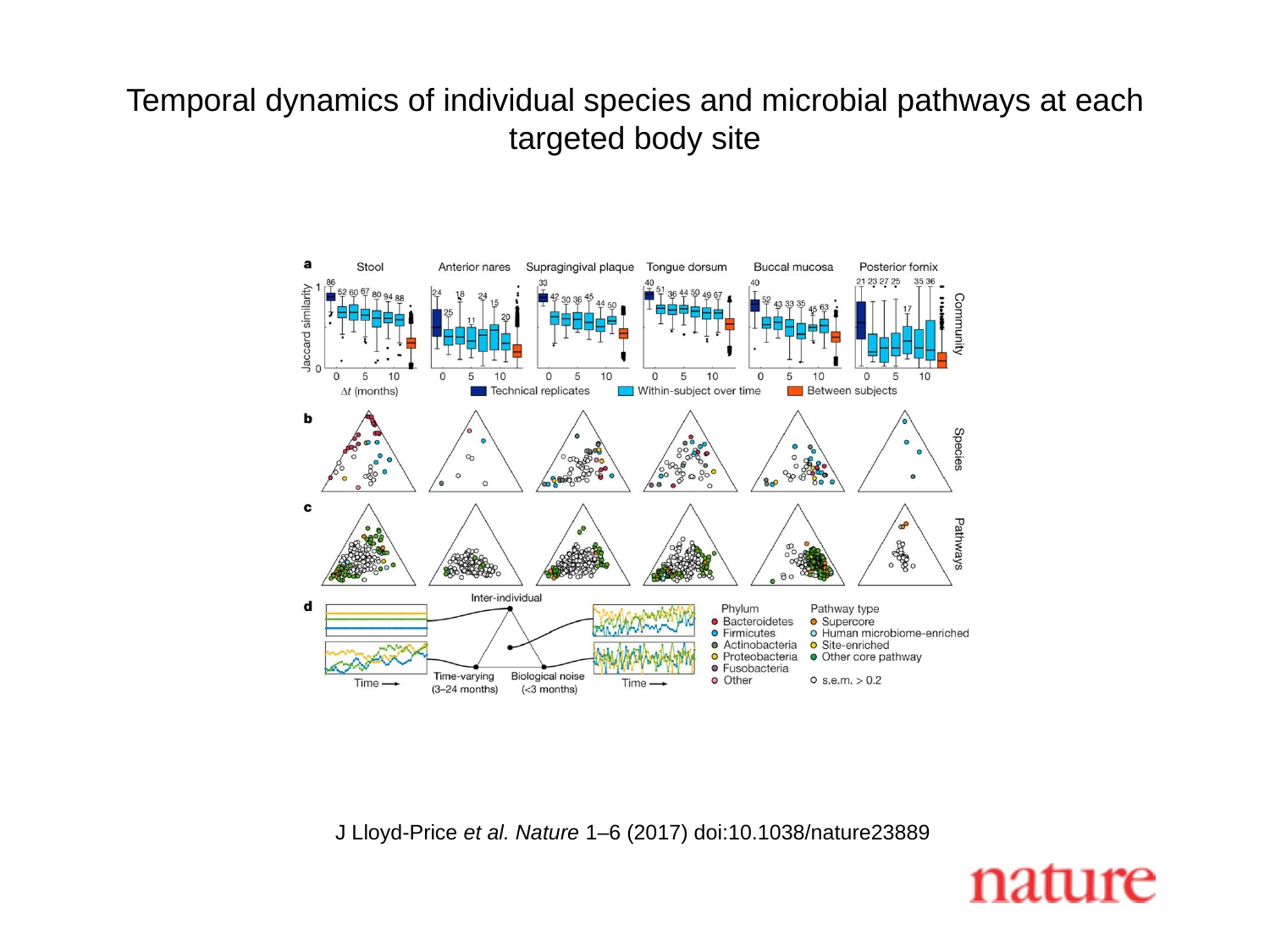

# Temporal dynamics of individual species and microbial pathways at each targeted body site
J Lloyd-Price et al. Nature 1–6 (2017) doi:10.1038/nature23889
